# Characterization of the *Populus* PLCP Gene Family and the Function of *PtrXCP2A* in Wood Formation

**DOI:** 10.3390/plants15203174

**Published:** 2026-10-16

**Authors:** Shenquan Cao, Xueke Wu, Xu Wang, Peng Guo, Fude Shang

**Affiliations:** College of Life Sciences, Henan Agricultural University, Zhengzhou 450046, China; caoshenquan@henau.edu.cn (S.C.); shangfude@henau.edu.cn (F.S.)

**Keywords:** *Populus trichocarpa*, *PLCP*, xylem fiber viability, wood formation, saccharification yield

## Abstract

Papain-like cysteine proteases (PLCPs) are a class of proteolytic enzymes that are involved in plant stress, immunity, growth and development processes. Compared to the extensive research on herbaceous plants, little is known about the *PLCPs* in trees, and the involvement of *PLCPs* in wood formation still needs to be confirmed. Here, 40 *PLCPs* were recognized in the genome of *Populus trichocarpa*, which can be divided into seven subfamilies with identified members: RD21, CEP, XCP, XBCP3, SAG12, RD19 and ALP. The genes in each subfamily exhibited similar gene structures, conserved motifs and domain compositions. Chromosome mapping analysis revealed that tandem and segmental duplications played crucial roles in expanding the *PLCP* family in *Populus*. Expression profiling, RT-qPCR verification and GUS activity analysis revealed that several *PtrPLCPs* were preferentially or highly expressed in the secondary xylem of the stem. Furthermore, we characterised *PtrXCP2A* overexpression in poplars and found that overexpression of *PtrXCP2A* accelerates the loss of xylem fiber viability, reduces fiber cell wall thickness and lignin content, but increases the saccharification yield of wood cell walls in transgenic poplar. These findings offer valuable information for future functional studies on *the PLCP* gene family, and an alternative gene for engineering lignocellulosic biomass for wood utilisation.

## 1. Introduction

Wood represents the most abundant lignocellulosic biomass in terrestrial ecosystems, primarily consisting of cellulose, hemicellulose and lignin, which collectively determine the biomechanical properties and industrial utility of wood. Cellulose, a linear polymer of β-1,4-linked glucose units, forms the crystalline microfibrillar skeleton that confers tensile strength to secondary cell walls (SCWs), and serves as the principal carbon source for pulp manufacturing and lignocellulosic biofuel production [1,2]. Hemicelluloses act as cross-linking agents between cellulose microfibrils, while lignin, a complex heterogeneous aromatic polymer, is impregnated into the SCW matrix to cement the polysaccharide network. Lignin deposition not only enhances the mechanical support and water transport capacity of stems, but also contributes to defense against biotic and abiotic stresses [1,3]. However, lignin is widely recognized as the major recalcitrant factor that impedes enzymatic hydrolysis of wood cell walls during industrial processing, including chemical pulping, forage digestion and cellulosic ethanol conversion. Therefore, dissecting the regulatory mechanisms underlying lignin biosynthesis and deposition during wood formation is of great importance for both fundamental tree developmental biology and forest biotechnology applications.

Lignin is synthesized through the conserved phenylpropanoid metabolic pathway. A series of core structural enzymes, including phenylalanine ammonia-lyase (PAL), 4-coumarate:CoA ligase (4CL), cinnamoyl-CoA reductase (CCR), cinnamate 4-hydroxylase (C4H), ferulate 5-hydroxylase (CAld5H), caffeoyl-CoA O-methyltransferase (CCoAOMT), and cinnamyl alcohol dehydrogenase (CAD), catalyze the stepwise generation of three monolignol monomers (p-hydroxyphenyl, guaiacyl and syringyl units), which are subsequently polymerized into lignin polymers in the apoplast by laccase (LAC) [3]. The transcriptional regulatory hierarchy of lignin biosynthesis has been well established: secondary-wall-associated NAC (SWN) transcription factors act as top master switches, activating a cascade of downstream MYB regulators (e.g., MYB021, MYB46, MYB074, WRKY19) that in turn directly induce the expression of SCW biosynthetic genes for lignin, cellulose (e.g., CESA4/7A/7B/8A/8B) and hemicellulose (e.g., GT47A-1, GT47C, CLSA1, GAUT12-A) [4,5,6,7,8,9]. Conventional genetic strategies typically reduce wood lignin content via direct suppression of key lignin biosynthetic genes. Nevertheless, such manipulations frequently lead to severe growth retardation and plant dwarfism, because impaired lignification of xylem vessels compromises long-distance water and nutrient transport, particularly in large woody species [3]. Notably, programmed cell death (PCD) refers to the genetically regulated, ordered cell death process during xylem development, characterized by nuclear degradation, tonoplast rupture and cellular content autolysis. Xylem fibers and vessels follow distinct developmental programs for SCW deposition and PCD patterns. Fiber-specific PCD modulation has been proven to effectively reduce total lignin content without compromising plant growth or vascular function, representing a more desirable strategy for wood quality improvement [3].

Papain-like cysteine proteases (PLCPs) are a major class of proteolytic enzymes widely distributed across viruses, bacteria, animals and plants [10]. In plants, PLCPs are synthesized as inactive proenzymes and become activated upon removal of the inhibitory propeptide. Accumulating studies have demonstrated that PLCPs participate in a wide range of biological processes, including seed germination, leaf senescence, abiotic stress responses, pathogen defense and developmental programmed cell death [11,12]. In the herbaceous model plant Arabidopsis, two xylem-specific PLCPs, *XCP1* and *XCP2*, have been characterized as key executors of micro-autolysis within the intact vacuole and mega-autolysis after tonoplast rupture during root xylogenesis [13]. Another KDEL-tailed PLCP, CEP1, localizes to both the endoplasmic reticulum and cell wall, and is involved in SCW thickening and cellular content clearance during stem xylem development [14]. In woody plants, fiber SCW thickening and PCD are tightly coupled sequential processes during secondary xylem development. The progression of fiber PCD defines the time window for SCW biosynthesis and deposition, which directly determines the final accumulation of lignin and cell wall polysaccharides [15,16]. A previous study on poplar aspartic proteases has shown that accelerated fiber PCD can shorten the SCW thickening period, reduce lignin content, and consequently enhance the saccharification efficiency of wood biomass [17]. However, genome-wide identification and functional characterization of the *PLCP* family in tree species remain scarce. Whether *PLCPs* are involved in wood formation by regulating fiber PCD and thus modulating lignin deposition remains unclear.

Poplars (*Populus* spp.) are fast-growing woody perennials that constitute a major source of lignocellulosic biomass for pulp and paper production, structural timber and bioenergy feedstocks worldwide. *Populus trichocarpa* has emerged as the predominant model tree species for investigating wood developmental mechanisms, benefiting from its well-annotated reference genome, established genetic transformation protocols and rich publicly available multi-omics datasets [18]. Although an early genome-wide survey of protease gene families in *Populus* was published, it was limited by the incomplete genome assembly of that era, and offered only limited insight into the classification, evolutionary dynamics and wood-associated expression profiles of the *PLCP* family [19]. Specifically, no functional evidence has yet linked *PLCPs* to the regulation of wood formation in any tree species. To fill this gap, we performed a comprehensive genome-wide identification and characterization of *PLCP* genes in *P. trichocarpa*, including phylogenetic classification, chromosomal distribution, gene duplication patterns, gene structure, conserved motifs and subcellular localization predictions. We further screened candidate *PtrPLCPs* associated with secondary xylem development by integrating multi-tissue expression profiles, stem developmental transcriptome data, SCW- and PCD-related *cis*-element analysis, and *promoter::GUS* activity assays. Finally, we functionally validated the role of *PtrXCP2A* via native-promoter-driven overexpression in poplar, and investigated its impacts on xylem fiber PCD, secondary cell wall deposition, wood composition and saccharification efficiency. This study provides a systematic foundation for exploring the functional diversification of the *PLCP* family in *Populus*, and identifies a novel candidate gene for the genetic improvement of lignocellulosic biomass for industrial utilization.

## 2. Results

### 2.1. Identification and Phylogenetic Analysis of PtrPLCPs

A total of 40 nonredundant sequences with complete open reading frames were identified in the *P. trichocarpa* genome (v 4.1). Then these potential sequences were confirmed with SMART and NCBI-CDD analyses by the presence of the complete peptidase_C1 domain (PF00112). Finally, these candidate PtrPLCPs were named PtrRD21A~C, PtrCEP1A~D, PtrXBCP3A~D, PtrSAG12A~R, PtrRD19A~E, PtrRDL1A~B, PtrALP2, PtrXCP1 and PtrXCP2A~B depending on their homology to *Arabidopsis* (Table S1). Other relevant information is shown in Table S2.

**Figure 1:**
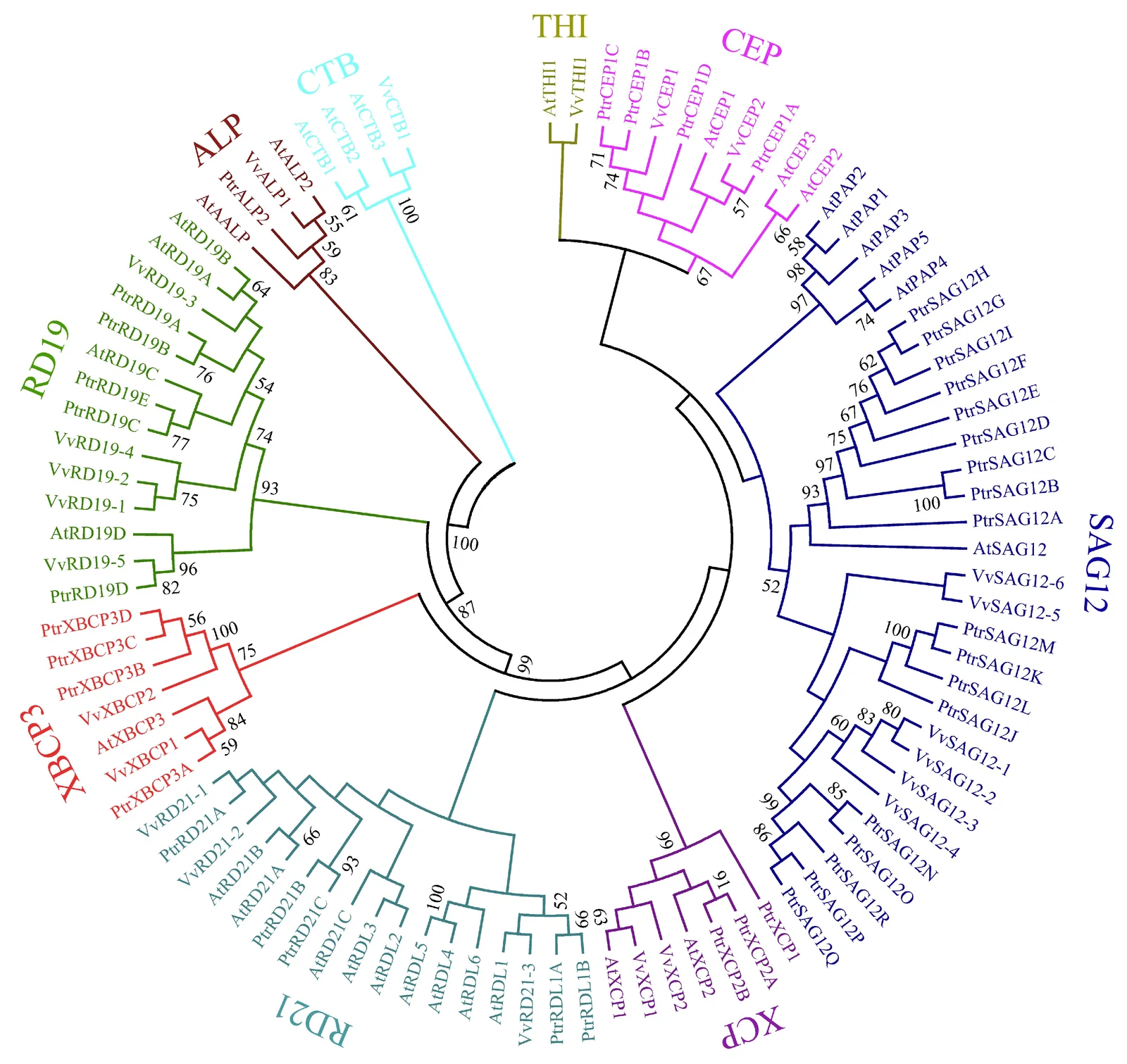
Phylogenetic subclassification of *P. trichocarpa*, *V. vinifera* and *A. thaliana* PLCPs. The full-length protein sequences of 40 PtrPLCPs, 23 VvPLCPs and 31 AtPLCPs were aligned with Clustal X, and the phylogenetic tree was constructed with the neighbor-joining method with MEGA 5.2 software under the default model with 1000 bootstrap replicates. All PLCPs were classified into nine subfamilies with different colors.

To investigate the phylogenetic relationship between PtrPLCPs and those of other plants, we performed a full-length protein sequence alignment among 23 VvPLCPs, 31 AtPLCPs and 40 PtrPLCPs (Table S3). The result showed that these PLCPs were separated into nine subfamilies (Figure 1), which is consistent with a previous study [11]. However, these PtrPLCPs were not evenly distributed among these nine subfamilies. SAG12 is the largest subfamily with 18 members, whereas the subfamilies CTB and THI do not contain any members. Both the RD21 and RD19 subfamilies contain five members, while the CEP and XBCP3 subfamilies each contain four members. The XCP and ALP subfamilies contain three and one members, respectively.

### 2.2. Conserved Motifs, Domains and Gene Structures of PtrPLCPs

**Figure 2:**
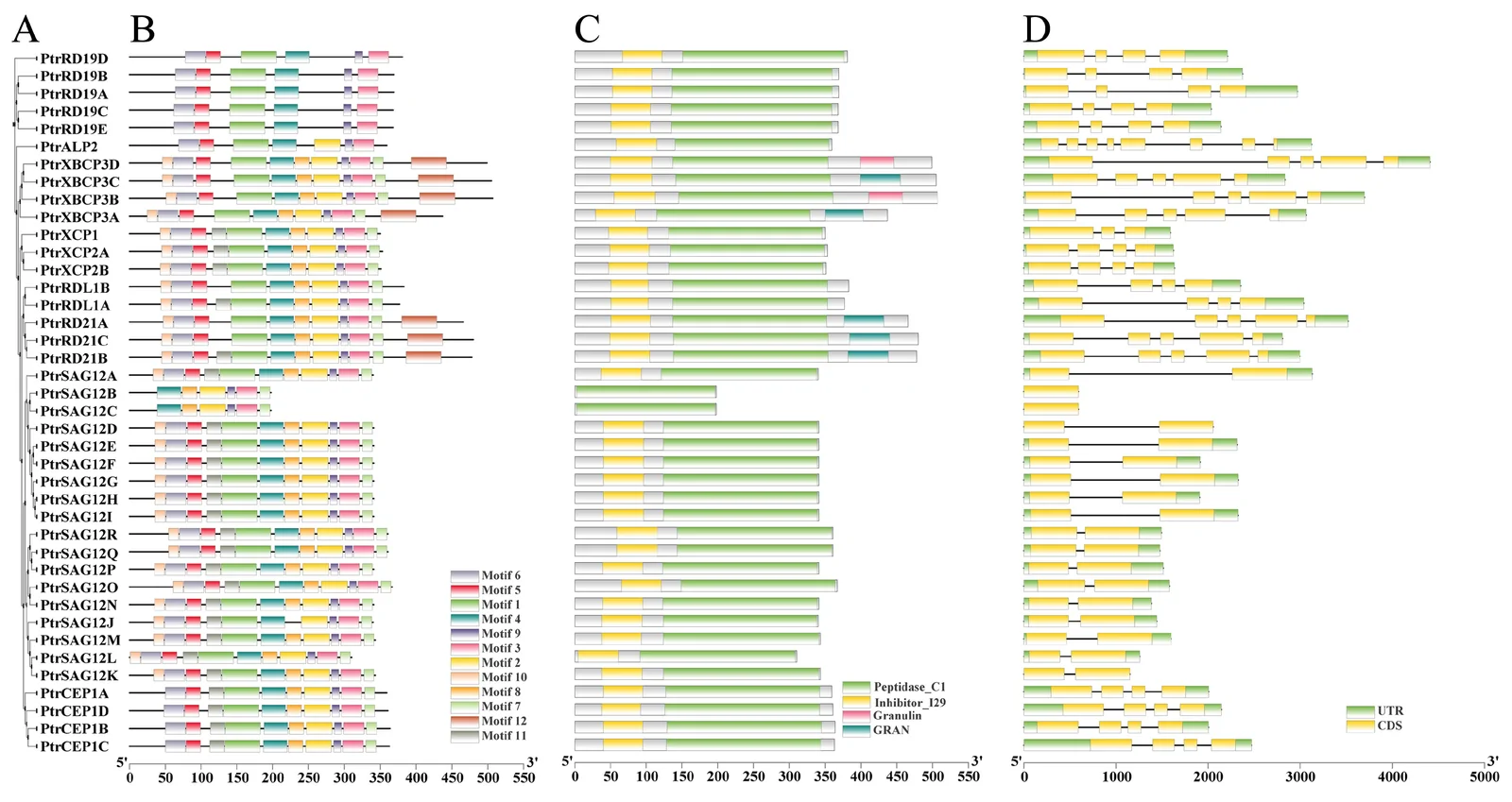
Phylogenetic tree, conserved motifs, domains and gene structures of 40 PtrPLCPs. (**A**) A phylogenetic tree of PtrPLCPs; (**B**) Distribution of twelve conserved motifs in PtrPLCPs; (**C**) Distribution of conserved domains in PtrPLCPs; (**D**) Gene structures of *PtrPLCPs*.

Conserved motifs and domains play important roles in the analysis and classification of gene families. To gain insight into the conserved and specific regions of these PtrPLCPs, we first analysed the phylogenetic relationship of PtrPLCPs as a reference (Figure 2A). Twelve highly conserved motifs were identified (Figure 2B). Detailed amino acid information for the motifs is shown in Figure S1. Most PtrPLCPs within the same subfamily showed similarly conserved motifs. Except for PtrSAG12B and PtrSAG12C, the remaining 38 PtrPLCPs contained motifs 1, 5, and 6, while all PtrPLCPs contained motifs 3, 4, and 9. Motif 12 was detected only in the RD21 and XBCP3 subfamilies. Multiple alignments were conducted to explore the conserved domains in PtrPLCPs (Figure 2C). As expected, the conserved Peptidase_C1 domain was present in all PtrPLCPs, and the inhibitor_I29 domain was present in all except PtrSAG12B and PtrSAG12C. In addition, the conserved granulin domain was present only in PtrRD21A~C and PtrXBCP3A~D.

Next, we analysed the gene structures of these 40 *PtrPLCPs* (Figure 2D). The closest genes shared similar structures regarding intron number or exon length; however, there was some variation among individual genes. Most genes from the subfamily SAG12 contained two exons, except *PtrSAG12B* and *PtrSAG12C*, both of which contained one exon. Members of subfamilies RD19 and CEP contained four exons, and those in subfamily XBCP3 contained five. Taken together, these results indicate a strong correlation between conserved motifs, conserved domains, and gene structures.

### 2.3. Chromosomal Location and Gene Duplication of PtrPLCPs

**Figure 3:**
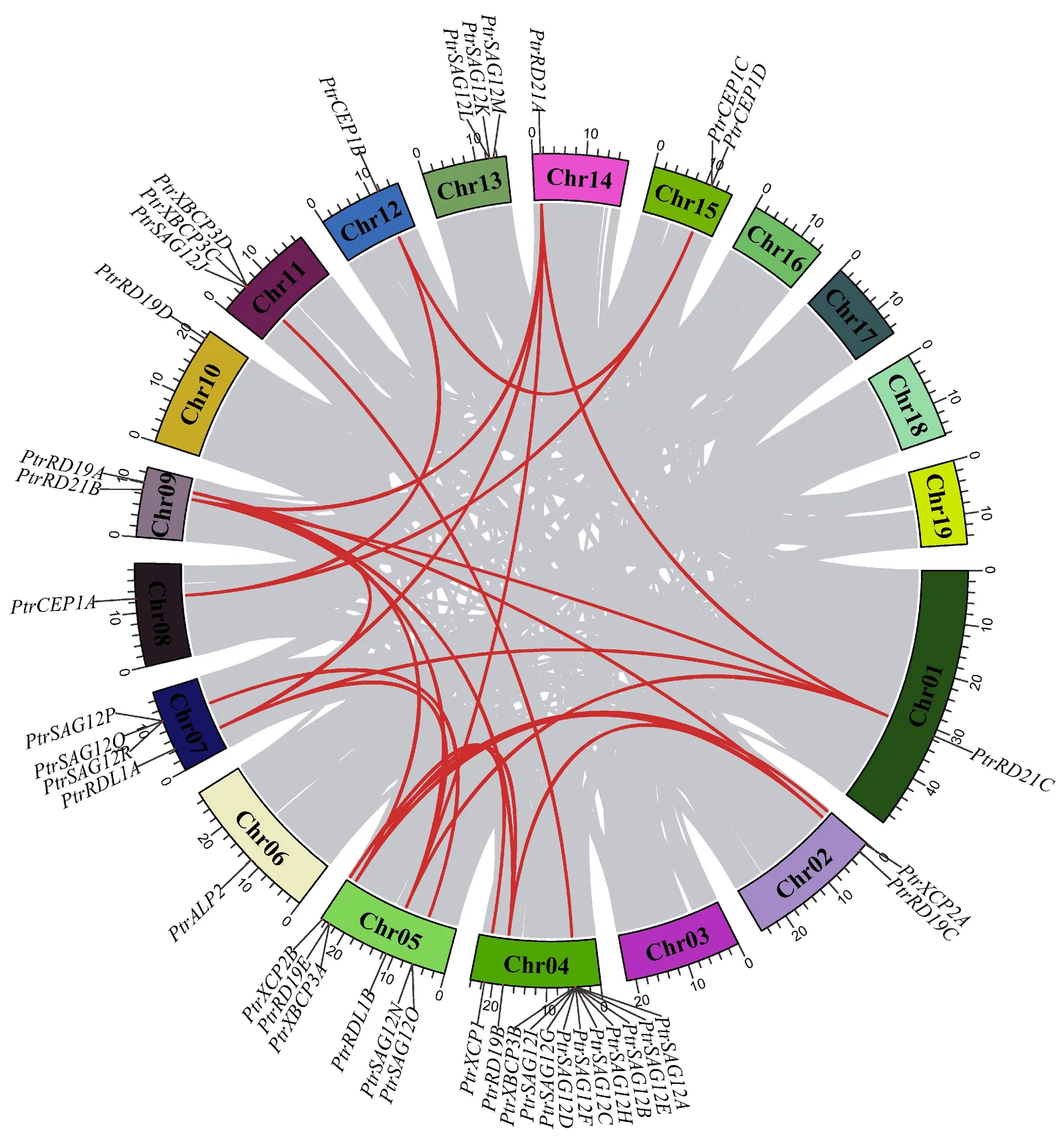
Chromosomal distribution and interchromosomal relationships of *PtrPLCP*s. Gray lines in the background represent the collinear blocks in the *P. trichocarpa* genome, and the red lines represent duplicated pairs among *PtrPLCP*s. The number in the block indicates the chromosome serial number, and the scale bar on the bottom indicates the chromosome lengths (Mb).

To visualise the chromosomal distribution of *PtrPLCPs*, we mapped these genes to the *P. trichocarpa* genome (Figure 3). The result showed that the chromosomal locations of these PtrPLCPs were scattered and uneven. Chromosome 04 had twelve *PtrPLCPs*, followed by chromosomes 05 and 07, which had six and four *PtrPLCPs*, respectively. Three *PtrPLCPs* were each located on chromosomes 11 and 13, and two *PtrPLCPs* were each located on chromosomes 02, 09 and 15, while chromosomes 01, 06, 08, 10, 12, and 14 contained only one member each. Subsequently, we analysed the expansion and evolution of *PtrPLCPs*, with seven gene clusters corresponding to 23 PtrPLCPs showing tandem duplicates and 22 gene pairs corresponding to 18 *PtrPLCPs* showing segmental duplications (Figure 3, Table S4). Notably, some subfamilies of *PtrPLCPs* have reciprocally duplicated genes, such as *PtrCEP1A/B/C*, *PtrRD19A/B/C/E*, *PtrRD21A/B/C* and *PtrRDL1A/B*, suggesting that they may share a common ancestral gene. Closely related genes of the subfamily SAG12 were mainly located on chromosomes 04, 05, 07, and 13, implying that the expansion of the SAG12 subfamily may have occurred via local or intra-chromosomal duplication. Although *PtrCEP1A*, *PtrXBCP3A*, *PtrXCP2A* and *PtrSAG12L* were located in the duplicated blocks, their duplicates (*Potri.010G051000*, *Potri.002G029800*, *Potri.009G168900* and *Potri.019G090100*, respectively) had lost the complete peptidase_C1 domain. Domain analysis via SMART and NCBI-CDD confirmed that these duplicated homologs have lost the complete peptidase_C1 domain during evolution. These findings show that both tandem and segmental duplications play vital roles in expanding the PtrPLCP family. In addition, we calculated the nonsynonymous substitution rate (Ka), synonymous substitution rate (Ks), and the Ka/Ks ratio of duplicated pairs. The results showed that all Ka/Ks values were <1.0 (Table S4), indicating that purifying selection influenced the *PtrPLCP* gene pairs after duplication.

### 2.4. Expression Patterns of PtrPLCPs

**Figure 4:**
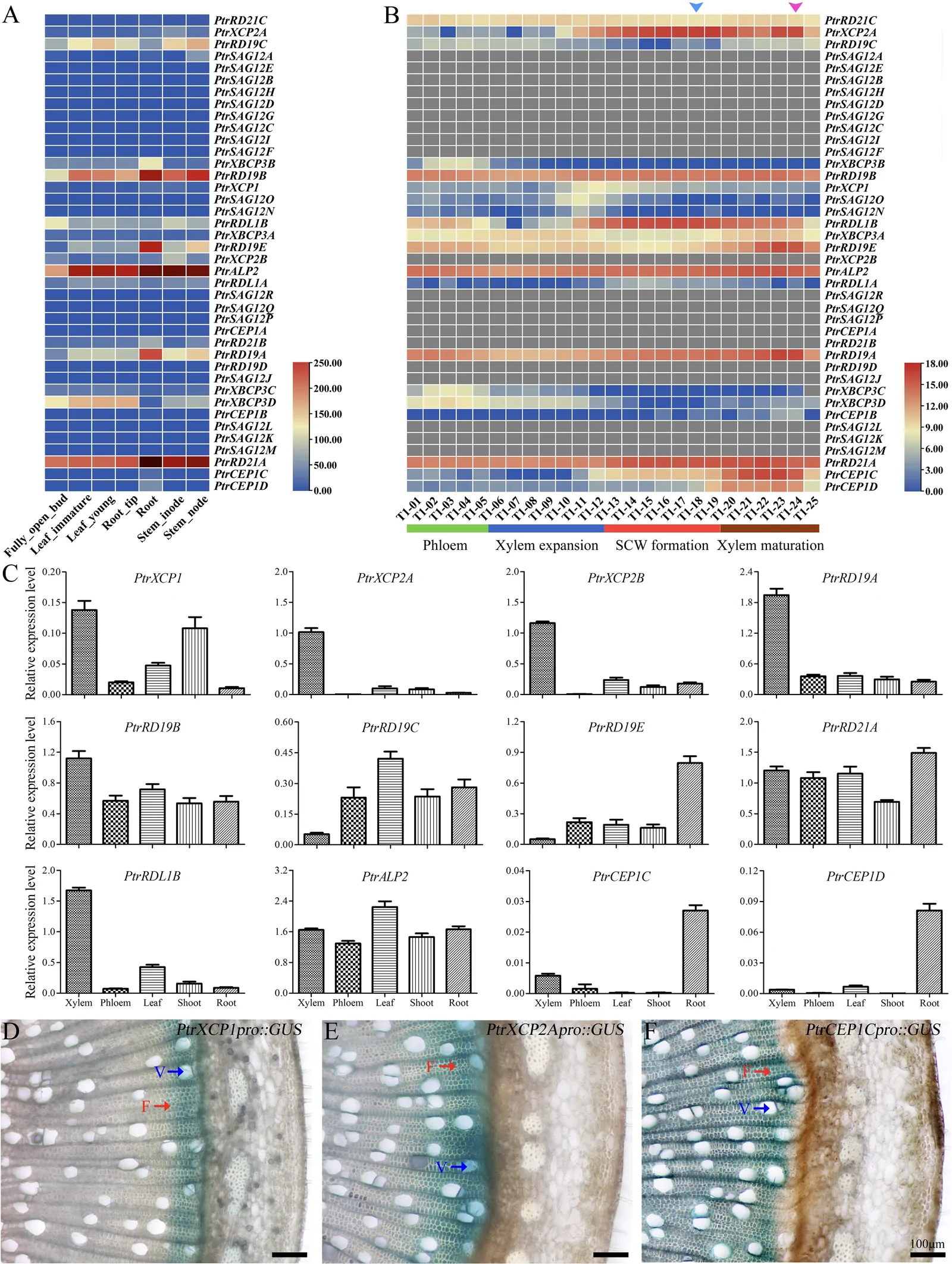
Expression pattern analyses of *PtrPLCPs*. (**A**) The fragments per kilobase of transcript per million mapped reads (FPKM) values of *PtrPLCPs* were retrieved from Phytozome (https://phytozome-next.jgi.doe.gov/, accessed on 17 August 2025). The color scale is shown at the lower right; (**B**) The RNA-seq data corresponding to forty *PtrPLCPs* were retrieved from Aspwood (http://aspwood.popgenie.org/aspwood-v3.0, accessed on 19 August 2025). The color scale at the lower right represents the variance-stabilized transformation values. The blue and pink triangles represent the end of vessel and fiber cell death, respectively; (**C**) Expression analysis of 12 selected *PtrPLCPs* in phloem, xylem, leaf, shoot and root. *PtrActin2* was used as an internal control, and all experiments were performed with three biological replicates, and error bars represent the standard deviation; (**D**–**F**) Promoter activity analyses of *PtrXCP1*, *PtrXCP2A* and *PtrCEP1C* in *P. trichocarpa* stem; V and F indicate the vessel and fiber, respectively.

To explore the potential roles of *PLCPs* in poplar, we examined the microarray files (from the Phytozome database) of *PtrPLCPs* in different tissues or organs of *P. trichocarpa*, including the buds, immature leaves, young leaf, root tip, root, internode, and node (Figure 4A). Most *PtrPLCPs* showed preferential expression patterns among these examined tissues according to their FPKM (Fragments Per Kilobase of exon model per Million mapped fragments) values. Subfamilies *RD21* and *RD19* in roots accounted for about 86% of total PLCP transcript abundance. In the internode, the subfamilies *RD21* and *RD19* were also the most abundant. Moreover, *PtrRD19A*, *PtrRD19E* and *PtrXBCP3B* were preferentially expressed in root; *PtrXCP2A* and *PtrXCP2B* were preferentially expressed in internode; *PtrRDL1B* was preferentially expressed in bud. In contrast, *PtrRD21A*, *PtrRD19B* and *PtrALP2* were highly expressed in all tissues and organs. The subfamily *SAG12* had the most members, but only a few were expressed (Figure 4A). We further investigated the microarray data (from the AspWood database) of *PtrPLCPs* in stems (Figure 4B). Among these genes, approximately a quarter of the members were highly expressed across the stem; *PtrXCP2A*, *PtrCEP1C* and *PtrCEP1D* were highly expressed in the mature xylem, which undergoes a transition from SCW formation to xylem PCD. *PtrXBCP3B/C/D* are primarily expressed in phloem, while *PtrXCP2A* is expressed throughout the xylem expansion zone up to the mature xylem stage, with the expression peak occurring during the mature xylem phase. To examine their preferential expression in the xylem of the stem, we validated the expression differences of 12 *PtrPLCPs* in various tissues or organs using RT-qPCR (Figure 4C). The expression patterns of most PtrPLCPs were similar to the microarray files. However, the transcript abundance of *PtrRD19A* and *PtrXCP1* was lower in root than in xylem or phloem. These errors may be caused by the different material protocols. In addition, *PtrRDL1B*, *PtrRD19A*, *PtrXCP2A* and *PtrXCP2B* were highly expressed in xylem, while *PtrCEP1C* and *PtrCEP1D* were preferentially expressed in roots. Their high expression in roots suggests their involvement in root xylem development, and their specific activity in stem xylem fibers supports their role as candidate regulators in wood formation. The diverse expression patterns suggest that *PtrPLCPs* are involved in multiple *Populus* developmental processes. To identify potential *PtrPLCPs* involved in wood development, we selectively examined the promoter activities of *PtrXCP1*, *PtrXCP2A* and *PtrCEP1C* in secondary xylem using a promoter::GUS approach (Figure 4D–F). Strong GUS signals were detected in the developing xylem of *PtrXCP1pro::GUS* lines and the mature xylem fibers of *PtrCEP1Cpro::GUS* plants. In contrast, *PtrXCP2A*-promoter-driven GUS signals were detected in the entire secondary xylem region. The different promoter-driven GUS expression patterns of *PtrXCP1*, *PtrXCP2A* and *PtrCEP1C* in stem were in accordance with the microarray data. Together, our results implied that *PtrXCP1*, *PtrXCP2A,* and *PtrCEP1C* might function in *Populus* wood development.

### 2.5. Analysis of the SCW-Related cis-Elements in Promoters of PtrPLCPs

**Figure 5:**
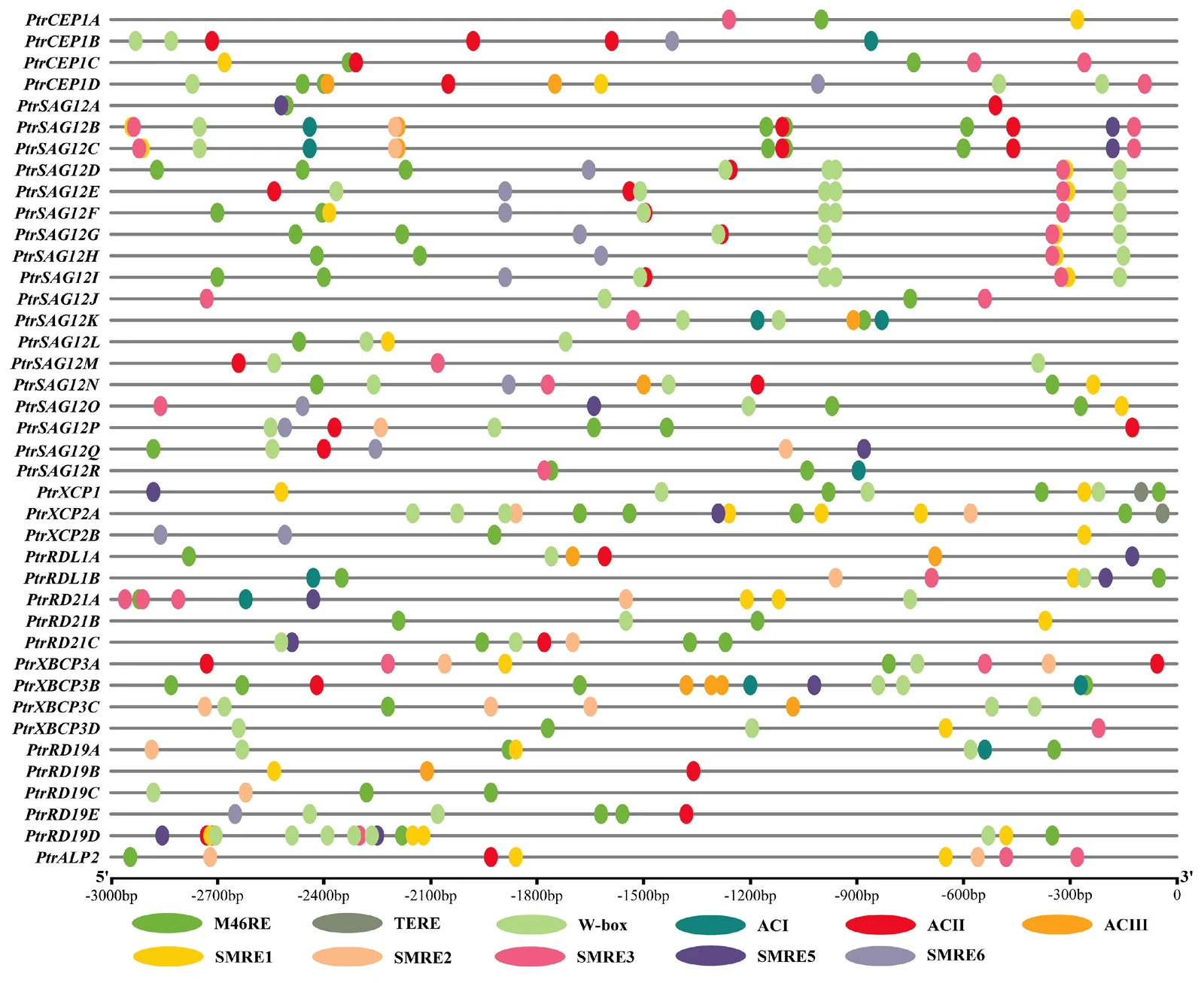
Analysis of SCW-related *cis*-elements in promoters of *PtrPLCP*s. The 3000 bp genomic DNA sequence from upstream of the translation start site (ATG) of each *PtrPLCP was* screened for SCW- and PCD-related *cis*-elements. SNBE was not detected, so it is not displayed. Different *cis*-elements were represented by differently colored diagrams.

Transcriptional regulatory networks coordinate SCW synthetic gene transcription during wood formation [9]. We surmised that *PtrPLCPs* might be the targets of SCW-regulated transcription factors (TFs). To further understand the potential function of these *PtrPLCPs*, we analysed the SCW-related core *cis*-elements in the promoters of *PtrPLCPs*, including SNBE, M46RE, TERE, W-box, AC and SMRE series (Figure 5). As expected, most promoter regions of these *PtrPLCPs* (except for *PtrCEP1B*, *PtrSAG12E*, *PtrSAG12M* and *PtrRD19B*) had the *cis*-element M46RE, which is necessary and sufficient for MYB46-responsive transcription. Similarly, except *PtrCEP1A*, *PtrCEP1C*, *PtrSAG12A*, *PtrSAG12R*, *PtrXCP2B*, *PtrRD19B*, and *PtrALP2*, all members contained the W-box *cis*-element, a binding site for WRKY TFs [20], as well as for the PtrMYB074–PtrWRKY19 dimer [5]. Nevertheless, the TE-related *cis*-element TERE exists only in the promoters of *PtrXCP1* and *PtrXCP2A*, and the key NAC-master-switch-related cis-element SNBE could not be found in any of the promoters of *PtrPLCPs*. Additionally, a series of AC and SMRE *cis*-elements involved in SCW biosynthetic activation were identified. Taken together, *cis*-element analysis of the upstream promoter regions revealed the presence of SCW- and PCD-related motifs, indicating that these *PtrPLCPs* may be transcriptionally regulated by SCW- and PCD-related transcription factors, providing preliminary clues for their potential roles in wood formation.

### 2.6. Overexpression of XCP2A Accelerates the Loss of Xylem Fiber Viability in Transgenic Poplar

**Figure 6:**
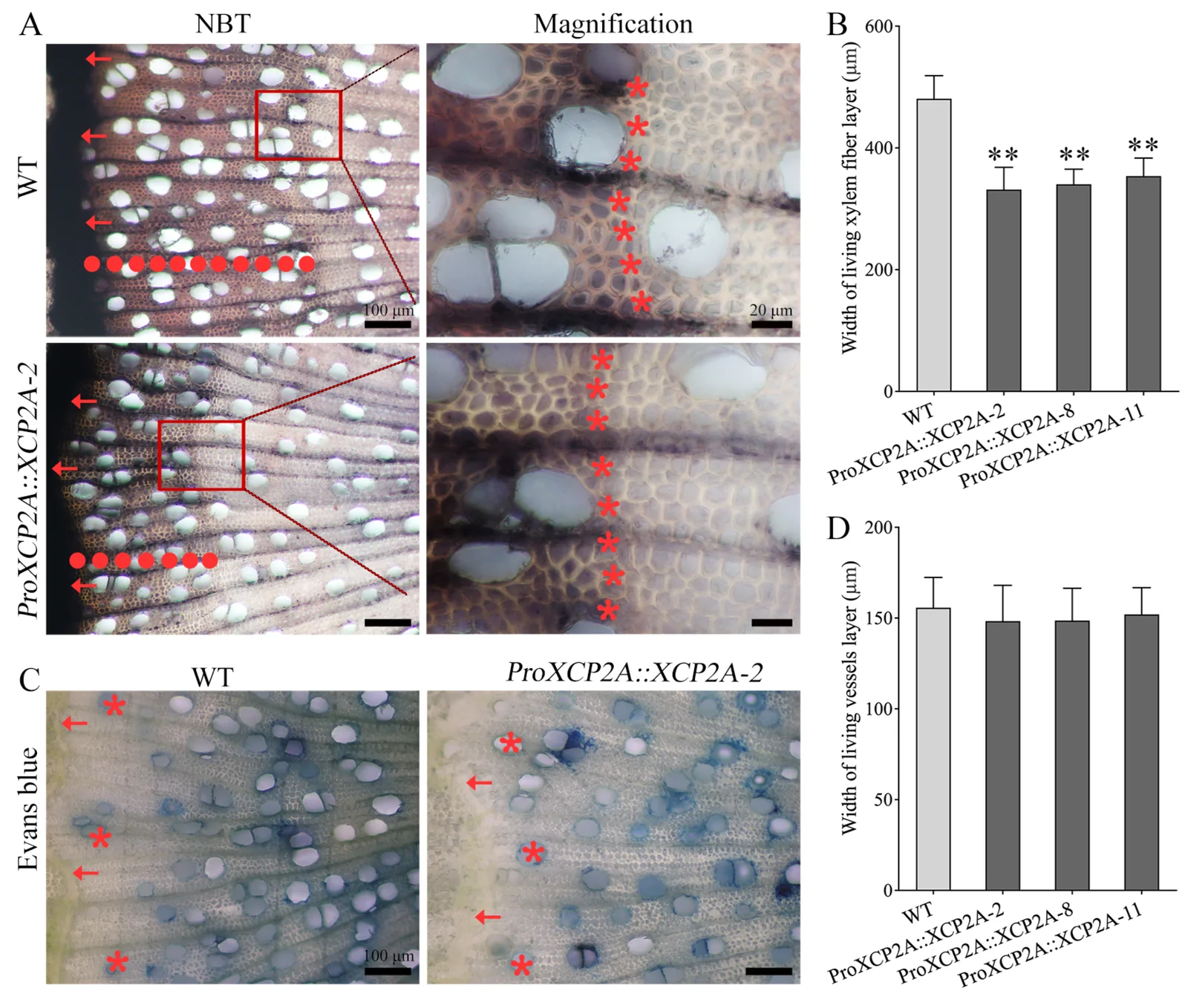
NBT and Evans blue staining of living xylem cells in *PtrXCP2A* overexpression plants. (**A**) Living xylem cells are black/purple, as partly indicated in red rectangles. Arrows point to the stem cambium, and a series of red points represent the width of living secondary xylem (fiber) layers; (**B**) The width of living secondary xylem layers; (**C**) The dead vessels have the ability to conduct water. Arrows point to the stem cambium, and a series of red asterisks represent the vessels with water-discharging capacity; (**D**) The width of the Evans-blue-stained conductive vessel region (the width of living xylem vessel layers). All experiments were performed with three biological replicates, and error bars represent the standard deviation. Asterisks represent significant difference from WT by one-way ANOVA with Dunnett’s test (** *p* < 0.01).

Gene expression patterns and functions were highly correlated. Based on the analysis of expression patterns and promoter cis-acting elements, we focused on *XCP2A*, which is mainly expressed in the late stage of xylem maturation and has a relatively high expression level (Figure 4A–C). Given the gene family expansion and potential functional redundancy of *PtrPLCPs*, we finally decided to adopt the native overexpression approach to verify the function of *PtrXCP2A*, and 13 independent transgenic poplar plants were obtained for *PtrXCP2A* overexpression. Three highly expressed lines (*ProXCP2A::XCP2A*-2/8/11) were measured by RT-qPCR, and retained for further analysis (Figure S2). Compared to WT plants, no significant growth phenotype differences were observed in *proXCP2A::XCP2A* lines, including tree height, stem diameter and cell sizes (Figure S3). Given that *PtrXCP2A* is mainly expressed in fibers, we hypothesize that it may play a role in the process of cell maturation. NBT staining showed that the living xylem fiber layers of *ProXCP2A::XCP2A* lines were reduced by 26.4–31.0% in comparison to WT plants, as the other lines (Figure 6A,B). In addition, we also examined the water-transport capacity of vessels in *proXCP2A::XCP2A* plants; Evans blue staining showed that the width of the Evans-blue-stained conductive vessel region was not significantly altered in *PtrXCP2A*-enhanced-expression lines (Figure 6C,D), indicating that enhanced expression of *PtrXCP2A* does not affect the formation of functional conductive xylem vessels. This may be related to the differential cascade regulation of vessel death from that in fibers. Together, our findings indicate that overexpression of *PtrXCP2A* is sufficient to accelerate the loss of xylem fiber viability in poplar.

### 2.7. Overexpression of XCP2A Alters SCW Synthesis and Enhances Saccharification Yield

**Figure 7:**
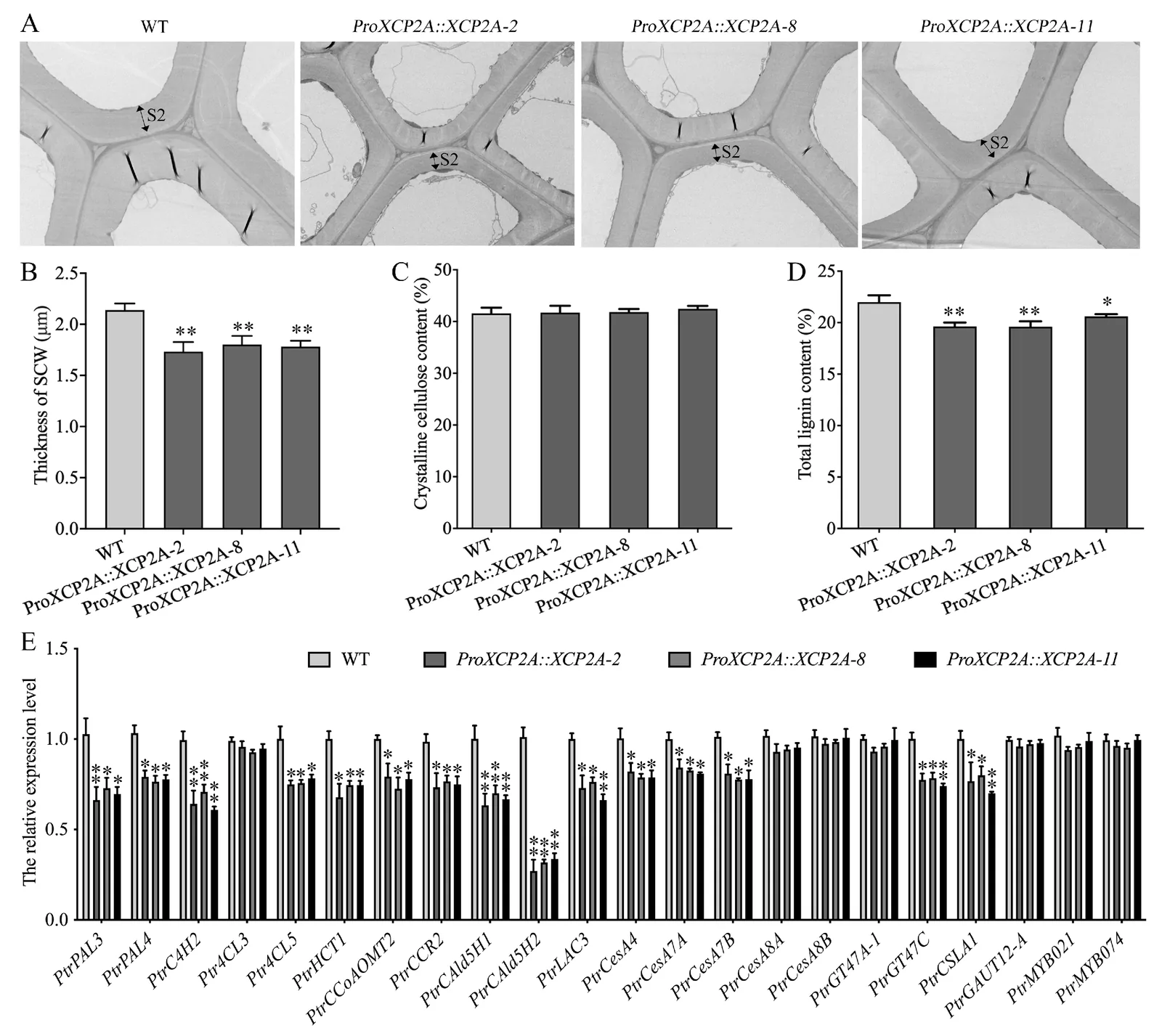
Overexpression of *XCP2A* alters wood SCW synthesis in poplars. The secondary cell wall (SCW) thickness (S2 layer) of fibers was recorded (**A**) and measured (**B**) from xylem of the WT and *XCP2A* transgenic plants. Starch-free wood was used to measure crystalline cellulose (**C**) and total lignin (**D**) contents. (**E**) Analysis of gene expression related to secondary xylem from the 20th internode in transgenic poplars. All experiments were performed with three biological replicates, and error bars represent the standard deviation. Asterisks represent significant difference from WT by one-way ANOVA with Dunnett’s test (* *p* < 0.05; ** *p* < 0.01).

Considering that fiber SCW synthesis and PCD of wood are coupled, we speculated that *PtrXCP2A* may be involved in the SCW synthesis of wood fibers. To test this hypothesis, we assessed the thickness of mature wood fibers in WT and *ProXCP2A::XCP2A* plants (Figure 7A). Particularly, the fiber wall thickness in *ProXCP2A::XCP2A* plants was reduced by 10.9–16.3%, compared to WT plants (Figure 7B). Moreover, the wood composition assay further revealed that the cellulose contents showed no significant change (Figure 7C), while the lignin contents decreased distinctly by 6.3–11.0% in *ProXCP2A::XCP2A* lines (Figure 7D). In addition, we also examined several enzyme genes and transcription factors related to lignin and cellulose synthesis. The results showed that the expression of most lignin biosynthetic genes was significantly downregulated, especially the key node gene *CAld5H* for the synthesis of S-lignin in fibers, while several cellulose and hemicellulose biosynthetic genes and some transcription factors showed no significant changes (Figure 7E). These findings revealed that overexpression of *PtrXCP2A* alters SCW biosynthesis during wood development in poplar.

**Figure 8:**
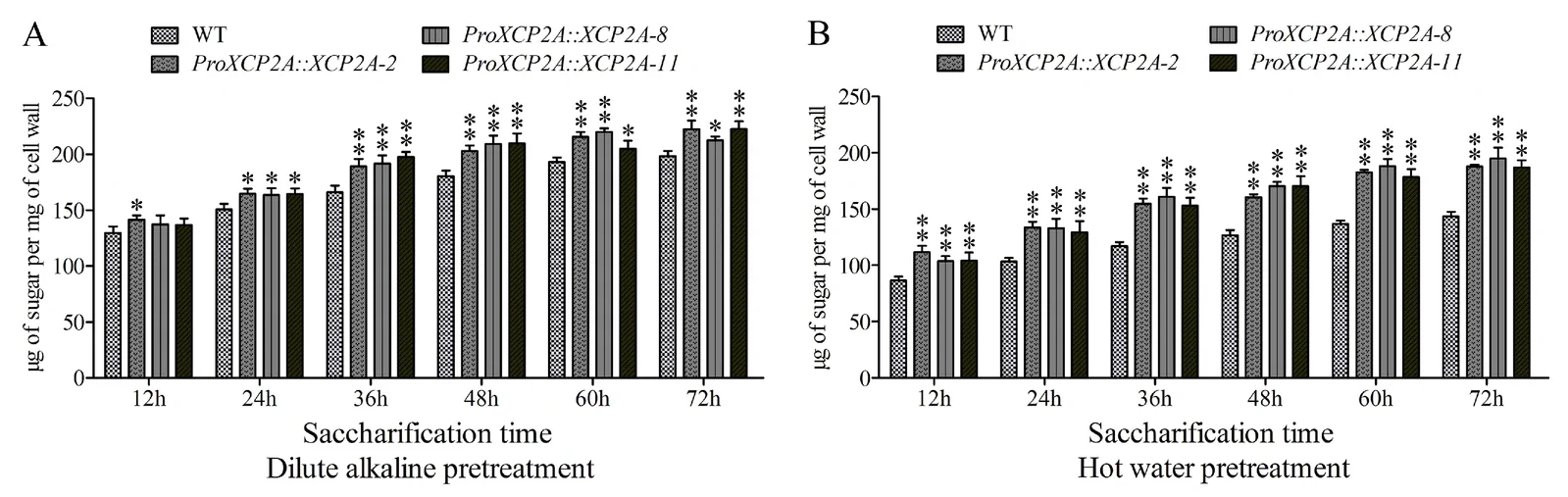
Saccharification yield of wood cell walls in *XCP2A* transgenic poplars. The amount of sugar released from wood powders of WT and *XCP2A* transgenic plants under dilute-alkali (**A**) and hot-water (**B**) pre-treatments, as well as the durations. All experiments were performed with three biological replicates, and error bars represent the standard deviation. Asterisks represent significant differences from WT by one-way ANOVA with Dunnett’s test (* *p* < 0.05; ** *p* < 0.01).

Since overexpression of *XCP2A* decreased the wood lignin content in *ProXCP2A::XCP2A* lines (Figure 7D), we further examined their wood cell wall digestibility with different treatments. The result showed that sugar release from *ProXCP2A::XCP2A* lines was faster than that from WT at each time point (Figure 8). Interestingly, the saccharification efficiency of wood cell walls under hot-water pre-treatment was significantly higher than that under dilute-alkali pre-treatment in *ProXCP2A::XCP2A* lines. For example, saccharification yields at 72 h were increased by 30.5–36.6% under hot-water pre-treatment and by 12.2–14.0% under dilute-alkali pre-treatment in *ProXCP2A::XCP2A* plants. Overall, these findings show that the overexpression of *XCP2A* enhances the saccharification efficiency of wood cell walls in *P. trichocarpa*.

## 3. Discussion

PLCPs are a class of proteolytic enzymes that are involved in leaf senescence, seed germination, PCD, stress response, plant development and immunity [10,11,12]. To the best of our knowledge, there have been no reports of the involvement of plant *PLCPs* in woody xylem development, and most previous functional studies or characterizations of PLCPs have focused on herbaceous plants, particularly Arabidopsis, maize, wheat, rice, soybean, tobacco, tomato, and barley [21,22,23,24,25,26,27,28,29,30,31], and have neglected the PLCP family in trees. In this study, we implemented a genome-wide analysis of the *PtrPLCP*s, including chromosomal location, gene duplication, structures, subcellular localisation, and glycosylation site, etc. Additionally, a set of *PtrPLCPs* (*PtrXCP1*, *PtrCEP1C* and *PtrXCP2A*, etc.) were proposed to participate in *Populus* wood development, as suggested by tissue expression patterns in stem, SCW- and PCD-related promoter *cis*-element screen and promoter-driven GUS activity analyses. We also demonstrate that *PtrXCP2A*, a xylem-preferential PLCP, accelerates the loss of xylem fiber viability, reduces SCW thickness and lignin content, and consequently enhances the saccharification efficiency of wood biomass. This work provides a comprehensive understanding of *PLCPs* in trees and identifies a novel regulator of wood fiber development with potential for lignocellulosic biomass engineering.

Compared with 31 PLCP members in *Arabidopsis*, the present work identified 40 PLCPs in the *P. trichocarpa* genome (Table S1). Considering the calculated ratio (1.4–1.6) of *P. trichocarpa* homologues per *A. thaliana* gene [18], the number of *PtrPLCPs* was slightly lower than expected relative to *A. thaliana*. Notably, *P. trichocarpa* has experienced three large-scale genome-wide duplications [18], but 55.0% of *PtrPLCPs* were located outside of any duplicated blocks, and some duplicates had lost the complete peptidase_C1 domain; presumably, this may be the main reason for the low number of *PLCPs* in *P. trichocarpa*. Even so, about 90% (36 of 40) of the *PtrPLCPs* were related to segmental or tandem duplications (23 genes are involved in tandem duplications, 18 genes are involved in segmental duplications, and five genes belong to both types), suggesting these *PtrPLCPs* may exist as functional redundancies. The most striking expansion occurs in the *SAG12* subfamily, which contains 18 members in poplar but only one in *Arabidopsis*. This remarkable expansion may be associated with the perennial life history of trees, which require more sophisticated regulatory networks for stress adaptation, seasonal senescence and long-term tissue homeostasis.

*PtrPLCPs* within the same subfamily showed similar localisation or motif patterns (Figure 2D; Table S2). For example, the canonical KDEL-tailed cysteine proteases were predicted to be located in the ER and are extensively found in collapsed tissues, particularly in cells undergoing PCD [32]. In this study, the KDEL-tailed PtrCEP1A/B/C/D form a distinct subgroup, which is consistent with CEP1/2/3 in *Arabidopsis* [21]. In the subfamily SAG12, four *PtrPLCPs* lacked UTRs (Figure 2D), as described in peanuts, grapes, castor beans, and physic nut [33,34,35]. It is possible that their common ancestral genes already possessed this specific gene structure. Although UTRs are not involved in amino acid translation, their presence can affect many aspects, including mRNA splicing, transport, degradation, and translation efficiency [36]. For members lacking the I29 prodomain (PtrSAG12B/C), we speculate that they may be atypical PLCPs whose activity does not require propeptide inhibition, or are regulated by other mechanisms, which remains to be functionally verified. Moreover, the RD21 and XBCP3 subfamilies uniquely possess a granulin domain, which is known to modulate protease activity and mediate protein–protein interactions, implying more complex post-translational regulation of their proteolytic activity [11]. In addition, tandem and segmental duplications have been the major driving forces for *PLCP* family expansion in poplar, and all duplicated pairs have undergone purifying selection (Figure 3; Table S4), suggesting that most duplicates have retained ancestral functions and may provide functional redundancy. This is consistent with the observation that the large SAG12 subfamily shows very low transcript abundance under normal growth conditions (Figure 4A,B). Overall, our work demonstrated the classification and evolutionary relationships of *PtrPLCPs*, and also raised the possibility that *PtrPLCPs* in the same subfamily may have correspondingly conserved functions.

Temporal and spatial expression patterns of genes provide further insights into the potential roles of *PtrPLCPs* in poplar. The *RD21* and *RD19* subfamilies are highly and broadly expressed across all tissues, suggesting they serve housekeeping proteolytic functions. In contrast, several members of the *XCP* and *CEP* subfamilies exhibit high expression in secondary xylem, indicating specialized roles in wood formation. Stem developmental gradient transcriptome data further resolved their spatial distribution: *PtrXCP1* is enriched in the expanding xylem zone, *PtrCEP1C* accumulates in mature xylem fibers, while *PtrXCP2A* is expressed across the entire secondary xylem zone. To explore the transcriptional regulation underlying these patterns, we filtered several SCW- or PCD-related core *cis*-elements from promoters of *PtrPLCPs*. Almost all promoters included the W-box and M46RE elements (Figure 5), both of which have been confirmed to be related to SCW formation or PCD processes. Interestingly, both stem developmental gradient RNA-seq data from the AspWood database and GUS staining (Figure 4D) analyses indicated that *PtrXCP2A* was expressed in vessels and fibers despite the presence of a TERE element in its promoter region, which is in charge of the synergistic expression of genes associated with PCD and SCW formation during vessel differentiation [7]. It is possible that additional regulatory regions exist in the *PtrXCP2A* promoter after gene duplication, therefore *allowing its expression* in fibers. In the *XCP2A*-overexpressing plants, xylem cell viability was distinctly reduced in transgenic *Populus*, suggesting that *PtrXCP2A* is involved in fiber PCD during wood formation. Nevertheless, we did not detect distinctly premature vessel death in secondary xylem of *XCP2A*-overexpressing poplars, which may be because the hydrolysis and autolysis processes in vessels were tightly followed by tonoplast rupture, which differs from those in fibers [16,37], and the accelerated PCD process in vessels was not sufficient to significantly increase in *XCP2A*-overexpressing plants. In addition, we acknowledge that *the possible involvement of PtrXCP2A* in fiber PCD is inferred from the viability phenotype and its homology to known PCD-related proteases. Direct evidence for regulation of PCD hallmark events (e.g., DNA fragmentation, tonoplast rupture) requires further validation by specific assays in future studies.

Functional validation by native-promoter-driven overexpression demonstrated that *XCP2A* is sufficient to accelerate the loss of xylem fiber viability, resulting in reduced fiber SCW thickness and decreased lignin content, without affecting overall plant growth or vessel function. Currently, only two types of proteases, the cysteine protease CEP1 and the aspartic protease AP17/45, are associated with PCD in xylem fibers [14,17]. However, the understanding of PCD in wood fibers is limited. The KDEL-tailed PLCP, CEP1, is located in the cell wall and involved in clearing xylem cellular contents during stem development in *Arabidopsis* [14]. Both *Populus* AP17 and AP45 contain two vacuolar sorting signals, a PSI domain and a C-terminal FAEA motif, both of which could result in them being ubiquitously located within the vacuole and provacuolar compartment [38], and overexpression of *AP17* promotes wood fiber PCD in secondary xylem [17]. Our findings identify PtrXCP2A as another type of protease involved in this process, representing the vacuolar papain-like cysteine protease class. Notably, *PtrXCP2A* acts differently from *CEP1*. Overexpression of *CEP1* in *Arabidopsis* did not alter xylem morphology, and instead upregulated the expression of cell wall biosynthetic genes, consistent with its role in clearing cellular contents during late PCD [14]. In contrast, *PtrXCP2A* overexpression reduced cell wall thickness and downregulated SCW biosynthetic genes (Figure 7), indicating that it acts at an earlier stage of xylem fiber maturation and viability loss. Additionally, this study only verified the overexpression of *PtrXCP2A* at the transcriptional level by RT-qPCR. Given that PLCPs are synthesized as inactive proenzymes, further validation via anti-FLAG Western blotting and activity-based protease assays is required to confirm the accumulation and activation of the target protein in xylem tissues.

The phenotype of *PtrXCP2A* overexpression is more similar to that of *AP17* overexpression in poplar, which also accelerates the loss of xylem fiber viability and reduces lignin content [17], suggesting that vacuolar proteases of different catalytic classes may act synergistically in the execution of fiber PCD. Presumably, the accelerated PCD process may well shorten the time window for SCW biosynthesis and deposition, leading to thinner SCW and reduced lignin accumulation. The observed downregulation of SCW-biosynthesis-related genes is likely a consequence of earlier termination of cellular metabolic activity, rather than a direct regulatory effect of *PtrXCP2A* on gene transcription. The mild increase in cellulose content is best explained as a relative compositional shift caused by the larger reduction in lignin, rather than increased absolute cellulose synthesis. This explanation is further supported by the saccharification results: hot-water pretreatment, which only removes labile hemicelluloses and is highly sensitive to lignin content, showed a much larger improvement in sugar release than dilute-alkali pretreatment, which already removes most of the lignin. In addition, from a biotechnological perspective, *XCP2A* represents a highly promising target for wood quality improvement. Conventional lignin reduction strategies, which directly repress key lignin biosynthetic genes, frequently cause severe growth retardation due to impaired vessel lignification and compromised water transport [3]. In contrast, *PtrXCP2A* modulates lignin content through fiber-specific viability regulation, without affecting plant growth or vessel function. This fiber-specific engineering strategy avoids the trade-offs associated with conventional approaches, and provides a candidate gene for the future genetic improvement of wood properties to improve lignocellulosic biomass for pulping and biofuel production. However, all the results are based on 4-month-old young poplar trees grown in a greenhouse. The long-term growth performance, wood mechanical properties, hydraulic safety, and biomass yield under field conditions of these trees still need to be evaluated. Together, our data suggest that overexpression of *XCP2A* alters wood fiber cell wall synthesis and PCD processes, and also offer an alternative gene for engineering lignocellulosic biomass for wood utilisation.

## 4. Materials and Methods

### 4.1. Identification of PLCP Genes in P. trichocarpa

Thirty-one AtPLCP sequences were retrieved from TAIR10 (http://www.arabidopsis.org/, accessed on 17 August 2025) for identifying potential *PLCPs* in *the P. trichocarpa* genome (https://phytozome-next.jgi.doe.gov/, accessed on 17 August 2025) v4.1 by BLASTP. The homology alignment parameters were set to an e-value ≤ 1 × 10^−15^ [39]. In addition to BLASTP, hmmsearch was performed against the *P. trichocarpa* proteome (v4.1) using the Peptidase_C1 (PF00112) HMM model from Pfam, with an e-value threshold of 1 × 10^−5^. The union of the two methods was deduplicated to obtain 43 candidate sequences. These available non-redundant longest candidate transcript/protein sequences (three redundant protein sequences were eliminated, including Potri.004G056500, Potri.007G076100 and Potri.004G056301) were further analyzed via NCBI-CDD (https://www.ncbi.nlm.nih.gov/Structure/cdd/wrpsb.cgi, accessed on 22 August 2025) and SMART (http://smart.embl-heidelberg.de/, accessed on 22 August 2025) tools to confirm the existence of the complete peptidase_C1 domain (PF00112). The partial or defective sequences (*Potri.002G184201*, *Potri.002G184300* and *Potri.014G109801*) were removed manually. ExPASy (https://web.expasy.org/compute_pi/, accessed on 23 August 2025) was used to calculate the theoretical isoelectric point and molecular weights. Five websites—LocTree3 (https://rostlab.org/services/loctree3/, accessed on 22 August 2025), ProtComp 9.0 (http://linux1.softberry.com/, accessed on 22 August 2025), Plant-mPLoc (http://www.csbio.sjtu.edu.cn/bioinf/plant-multi/, accessed on 22 August 2025), SherLoc2 (https://abi-services.informatik.uni-tuebingen.de/sherloc2/webloc.cgi, accessed on 22 August 2025), and WOLF PSORT (https://www.genscript.com/wolf-psort.html, accessed on 22 August 2025)—were used for localisation prediction, and the final results were presented according to the majority.

### 4.2. Phylogenetic Analysis

As previously described, multiple sequence alignment and phylogenetic analyses of PLCPs were performed [40]. Forty PtrPLCPs, 23 VvPLCPs and 31 AtPLCPs were aligned using Clustal X software (v1.83). Phylogenetic trees of PLCPs were constructed using MEGA 5.2 software with the Neighbor-Joining method with 1000 bootstrap replicates.

### 4.3. Genomic Organisation, Duplication, Gene Structure and Conserved Motifs Analyses

The corresponding *PLCP information was* retrieved from the genome annotation files of *P. trichocarpa*. Gene location, duplication events, and collinearity analyses of *PLCPs* were performed as previously described [41]. For the criteria, tandem duplication: homologous gene pairs located on the same chromosome, less than 100 kb apart, with no more than one intervening gene; segmental duplication: homologous gene pairs located within genomic collinear blocks. The diagram was drawn using MCScanX software in TBtools-II (v2.441). The GSDS tool (http://gsds.gao-lab.org/, accessed on 22 August 2025) was used to analyse gene structure [42]. The MEME tool (https://meme-suite.org/meme/, accessed on 22 August 2025) was used to identify the conserved motifs [43]; the maximum number of motifs was set to 12, the width was 11–50 amino acids, and the default values were used for the others.

### 4.4. SCW-Related Cis-Elements Screen

The 3 kb promoter sequences of *PtrPLCPs* were retrieved and scanned for the SCW-related core *cis*-elements (both sense and antisense strands of the promoter sequences were scanned for each motif): SNBE (WNNYBTNNNNNNNAMGNHW), TERE (CTTNAAAGCNA), M46RE (RKTWGGTR), W-box (TTGACT), ACI (ACCTACC), ACII (ACCAACC), ACIII (ACCTAAC), SMRE1 (ACCAAAT), SMRE2 (ACCAACT), SMRE3 (ACCAAAC), SMRE5 (ACCTAAT) and SMRE6 (ACCTACT). These core *cis*-elements have been confirmed to participate in xylem SCW biosynthesis and (or) PCD processes [4,5,6,7,8]. Diagrams of these SCW-related *cis*-elements in the promoters of *PtrPLCPs* were drawn by TBtools-II software (v2.441) [44].

### 4.5. Plant Materials, Growth and Agrobacterium Tumefaciens-Mediated Transformation

*P. trichocarpa* (Nisqually-1) was used and maintained in the Henan Agricultural University greenhouse in China. The detailed conditions for tissue cultivation and genetic transformation were described in our previous work, and the samples were collected from shoot, phloem, xylem, leaf and root [45]. The AspWood database contains high-resolution RNA-seq data across the stem developmental gradient of *Populus tremula*. We mapped *P. tremula* transcripts to *P. trichocarpa* v4.1 gene models via reciprocal best BLASTP alignment with an e-value threshold of 1 × 10^−10^.

Approximately 3 kb promoter regions (genomic sequence upstream of the translation start site ATG) of *PtrXCP1, PtrXCP2A*, and *PtrCEP1C* were amplified and cloned into the pENTR/D-TOPO (Gateway, Invitrogen, Carlsbad, CA, USA) vectors, respectively. The correctly sequenced entry vectors were interchanged with the plant expression vector pGWB3 and then transformed into the *Agrobacterium tumefaciens* strain GV3101. For *the XCP2A* overexpression construct, the native promoter (as above) and CDS (coding sequence) of *PtrXCP2A* were amplified and spliced to construct the vector pGWB10-*PtrXCP2A*-*FLAG*. Then the *ProXCP2A::XCP2A-FLAG* construct was introduced into GV3101. The primers used in this study are listed in Table S5.

### 4.6. RNA Extraction and RT-qPCR Analyses

Quick-frozen poplar tissues were ground into a fine powder, and total RNA of different tissues was extracted using the modified cetyltrimethylammonium bromide (CTAB) method as previously described [46]. RNA quantity was measured by a NanoDrop 2000 spectrophotometer (Thermo, Waltham, MA, USA), followed by cDNA synthesis [47] and RT-qPCR analyses (TaKaRa, Beijing, China). The comparative Ct (2^−Δ^*^Ct^* and 2^−ΔΔ^*^Ct^*) methods were used to calculate gene expression levels [48], with three biological replicates and three technical replicates per biological replicate. RNA integrity, DNase treatment, reverse transcription, qPCR system, primer amplification efficiency, fragment amplification length and amplification program followed the instructions for execution (TaKaRa, Beijing, China). *PtrActin2* was used as an internal control [17], and all primers used are listed in Table S5.

### 4.7. GUS Staining and Cell Viability Analyses

Promoter activity analysis of the transgenic poplars was carried out as previously described [17]. Four independent transgenic lines (three independent plants per line) for each promoter were used for the activity analysis, and the cross-sections of stems were collected for GUS staining.

Xylem cell viability and vessel death were analysed as described previously [49]. For the width analysis of living xylem fiber layers, the cross-sections of the 20th internode from 4-month-old wild-type (WT) and overexpression poplars were collected and dyed in 0.1% NBT solution. For the death analysis of xylem vessels, note that a vessel becomes hollow as it matures and dies, and is then used to transport water. The poplar twigs were inserted in 1% Evans blue solution, and the cross-sections of the 15th internode were collected and recorded by a BX43 upright microscope as above (Olympus, Hachioji-shi, Japan). The boundary selection criteria start from the first fully dye-filled vessel layer inward from the cambium and end at the innermost functional vessel, and the width of this region is measured. Three sections per plant were analyzed, with at least three biological replicates included in the experiment.

### 4.8. Morphological Feature Analyses

The poplar plantlets were planted in soil for phenotypic analysis. The plant heights were measured monthly from the shoots to the fixed reference points (20 cm above the soil), as were the stem diameters. The sizes of xylem fibers and vessels were measured as described previously [50]. The peeled 22nd internode was macerated in an acid dissociation solution for 4 h. After gentle rinsing and mechanical shaking, the suspension cells were drawn and mixed with equal volumes of acid fuchsin solution (0.1%), then photographed as above for subsequent measurement. At least six independent plants per line were used. Different internodes correspond to distinct developmental stages of wood formation. The 12th internode with active secondary wall deposition is suitable for TEM observation; the 15th internode with completed vessel differentiation is suitable for Evans blue staining; the 20th internode at the fiber maturation transition stage is suitable for NBT staining and gene expression; the 22nd internode with completed cell elongation is suitable for cell maceration; the basal stem with accumulated mature xylem is suitable for composition and saccharification assays. This is a standard sampling strategy in wood development studies.

### 4.9. Xylem Fiber Morphology and Wood Composition Assay

Transmission electron microscopy analysis was performed as previously described [17]. The 12th internode of WT and transgenic plants was cut into lengths of 2–3 mm and fixed in 0.1 M PBS buffer (pH 7.4) containing 2.5% (*w*/*v*) glutaraldehyde and 4% (*w*/*v*) paraformaldehyde (Sigma-Aldrich, Burlington, MA, USA). The samples were sequentially washed, dehydrated, immersed, sectioned, and dyed, and then the xylem fiber morphology analysis was performed in an HT-7700 electron microscope (HITACHI, Tokyo, Japan). Three sections per tree were analyzed (15 morphologically intact fiber cells were randomly selected in each section, and the S2 layer thickness of radial walls was measured and recorded by ImageJ2), with at least three biological replicates. For the wood composition assay, the peeled basal stems from 4-month-old trees were dried at 55 °C for grinding. Then the powders were washed successively with 70% ethanol, chloroform/methanol (1:1) solution and acetone, and finally the insoluble residues were air-dried and prepared as the cell wall materials for the composition and saccharification assays. Lignin determination used the acetyl bromide method, and crystalline cellulose determination used the anthrone colorimetric method; results were expressed as a percentage of extractive-free cell wall dry weight. The detailed experimental process can be found in our previous research [51].

### 4.10. Saccharification Assays

Saccharification efficiency of wood cell wall materials was quantified following a procedure adapted from previous protocols [2]. Briefly, wood samples were first dried and pulverized via ball milling, and the resulting fine powder was transferred to centrifuge tubes for subsequent pretreatment. For hot-water pretreatment, samples were mixed with ultrapure water, while the dilute-alkaline pretreatment group was incubated in a 1% (*w*/*v*) NaOH solution. To initiate enzymatic hydrolysis, an appropriate volume of 83 mM sodium citrate buffer (pH 6.2) was added to each system, bringing the final enzyme concentrations to 4.4% (*w*/*w*) cellulase R10 (Yakult Pharmaceutical Industrial Co., Ltd., Kunitachi-shi, Japan) and 0.44% (*w*/*w*) β-glucosidase (Yuanye Bio-Technology, Shanghai, China). All reaction systems were incubated at 50 °C for a series of preset time intervals, and the concentration of reducing sugars released into the supernatant at each time point was determined using the 3,5-dinitrosalicylic acid colorimetric method.

### 4.11. Data Analysis

All experiments were performed with three biological replicates, and error bars represent the standard deviation. One-way analysis of variance (ANOVA) followed by Dunnett’s post hoc test was used for multiple comparisons between wild-type and transgenic lines [52]. Normality of data distribution and homogeneity of variance were tested by the Shapiro–Wilk test and Levene’s test, respectively. All data met the assumptions of parametric tests.

## 5. Conclusions

In summary, this study provides a comprehensive genome-wide analysis of the PLCP gene family in *Populus*, revealing its evolutionary expansion, structural diversification and expression landscape in relation to wood development. Functional characterization identifies PtrXCP2A as a novel regulator of secondary xylem fiber viability and cell wall deposition, with potential application for lignocellulosic biomass improvement. These findings not only advance our understanding of PLCP family evolution and function in trees, but also provide new genetic tools and a conceptual framework for future engineering of wood properties.

## Data Availability

The genome sequences and gene annotation data of *Populus trichocarpa* v4.1 are available on Phytozome (https://phytozome-next.jgi.doe.gov/, accessed on 17 August 2025). All other data supporting the findings of this study are included within the article and its Supplementary Materials.

## Supplementary Materials

The following supporting information can be downloaded at https://www.mdpi.com/article/10.3390/plants15203174/s1, Figure S1: Conserved motifs identified by the MEME online tool in 40 *PtrPLCPs*; Figure S2: Transcriptional levels of *XCP2A* in xylem of transgenic plants. Expression analysis of *XCP2A* in WT and *XCP2A*-OE poplars. *PtrActin2* was used as an internal control. All experiments were performed with three biological replicates, and error bars represent the standard deviation. Asterisks indicate significant differences from WT by one-way ANOVA (* *p* < 0.05; ** *p* < 0.01); Figure S3: Characterization of the *XCP2A* transgenic poplars. (A) Morphology of 3-month-old WT and *XCP2A* transgenic poplars. (B) Statistical analysis of tree height, stem diameter, xylem fiber length and width and xylem vessel element length and width in WT and *XCP2A* transgenic poplars. At least six plants of each line were analysed; Table S1: *PLCP* gene family in *Populus trichocarpa*; Table S2: The predictable subcellular localisation of 40 PtrPLCPs; Table S3: The sequences of PLCPs from *P. trichocarpa*, *V. vinifera* and *A. thaliana*; Table S4: The Ka/Ks values of collinearity pairs of the *PtrPLCPs*; Table S5: The primers used in this study.
