# Supplementary material for: Characterization of the Populus PLCP Gene Family and the Function of PtrXCP2A in Wood Formation
Source: Plants (Basel). 2026 Oct 16;15(20):3174. doi: 10.3390/plants15203174 (PMC13708765; doi:10.3390/plants15203174)
Supplement: Supplementary file 1 [file plants-15-03174-s001.zip › Table S1.pdf]

Table S1. ***PLCP*** **gene family in** ***Populus trichocarpa***

Gene locus	Gene symbol	Protein length (aa)	Isoelectric point (PI)	Molecular weight (MW)	N-/O-glycosylation sites[(a)]	Subcellular Localization[(b)]
Potri.001G302100	PtrRD21C	479	5.30	52836.33	YES/YES	Ext
Potri.002G005700	PtrXCP2A	352	5.23	39393.42	YES/NO	Vac
Potri.002G028700	PtrRD19C	367	5.48	40333.30	YES/YES	Ext
Potri.004G055900	PtrSAG12A	339	7.95	37402.56	YES/YES	Ext
Potri.004G056000	PtrSAG12E	340	5.50	37622.07	YES/YES	Ext
Potri.004G056050	PtrSAG12B	197	4.49	20834.94	NO/YES	ER
Potri.004G056100	PtrSAG12H	340	5.21	37650.14	YES/YES	Ext
Potri.004G056200	PtrSAG12D	340	5.41	37546.08	YES/YES	Ext
Potri.004G056258	PtrSAG12G	340	5.31	37578.07	YES/YES	Ext
Potri.004G056308	PtrSAG12C	197	4.64	20715.88	NO/YES	Not clear
Potri.004G056316	PtrSAG12I	340	5.31	37592.10	YES/YES	Ext
Potri.004G056366	PtrSAG12F	340	5.42	37575.06	YES/YES	Ext
Potri.004G057700	PtrXBCP3B	506	4.79	56126.94	YES/YES	Not clear
Potri.004G160300	PtrRD19B	368	5.54	40282.35	YES/YES	Ext
Potri.004G207600	PtrXCP1	349	5.44	38925.78	YES/YES	Vac
Potri.005G088600	PtrSAG12O	366	5.92	40034.13	YES/YES	Not clear
Potri.005G089100	PtrSAG12N	340	5.55	37280.83	YES/YES	Ext
Potri.005G141600	PtrRDL1B	382	5.01	42532.65	NO/YES	Ext
Potri.005G232900	PtrXBCP3A	436	6.59	47919.23	YES/YES	Ext
Potri.005G234000	PtrRD19E	367	6.01	40273.46	YES/YES	Ext
Potri.005G256000	PtrXCP2B	350	5.31	38995.95	YES/YES	Vac
Potri.006G141700	PtrALP2	358	6.26	39352.46	YES/YES	Vac
Potri.007G047600	PtrRDL1A	376	5.70	42222.36	YES/YES	Ext
Potri.007G075300	PtrSAG12R	360	6.15	39418.61	YES/YES	Ext
Potri.007G075900	PtrSAG12Q	360	6.15	39440.56	YES/YES	Not clear
Potri.007G076000	PtrSAG12P	340	5.71	37375.96	YES/YES	Ext
Potri.008G183100	PtrCEP1A	358	5.82	40189.01	NO/YES	ER
Potri.009G098100	PtrRD21B	477	5.39	52446.00	YES/YES	Ext, ER
Potri.009G121300	PtrRD19A	368	5.81	40440.65	YES/YES	Vac
Potri.010G228400	PtrRD19D	380	6.22	41625.61	YES/YES	Ext
Potri.011G064900	PtrSAG12J	339	4.97	36997.37	YES/YES	ER
Potri.011G066800	PtrXBCP3C	504	4.75	55945.86	YES/YES	Ext
Potri.011G066900	PtrXBCP3D	498	4.87	55084.96	YES/YES	Ext
Potri.012G090900	PtrCEP1B	363	6.05	40417.59	YES/YES	ER
Potri.013G118200	PtrSAG12L	309	4.85	33951.63	YES/YES	Not clear
Potri.013G118400	PtrSAG12K	342	4.78	37816.31	YES/YES	Ext
Potri.013G126100	PtrSAG12M	342	5.35	37965.53	YES/YES	Ext
Potri.014G024100	PtrRD21A	465	5.42	51412.68	YES/YES	Ext
Potri.015G087400	PtrCEP1C	362	5.93	40461.51	YES/YES	ER
Potri.015G087500	PtrCEP1D	360	6.67	40209.32	YES/YES	ER

[a] Protein N- and O-glycosylation sites were predicted by NetNGlyc 1.0 and NetOGlyc 4.0 server, respectively. "YES" represented at least exist one glycosylation sites (positive) and "NO" represented negative results.

[b] Protein subcellular localization was predicted by softwares (Plant-mPLoc, LocTree3, ProtComp 9.0, WOLF PSORT, and SherLoc2), and the final results were presented according to the majority, otherwise not clear. ER, endoplasmic reticulum; Ext, extracellular; Vac, vacuole.
