# Supplementary material for: Characterization of the Populus PLCP Gene Family and the Function of PtrXCP2A in Wood Formation
Source: Plants (Basel). 2026 Oct 16;15(20):3174. doi: 10.3390/plants15203174 (PMC13708765; doi:10.3390/plants15203174)
Supplement: Supplementary file 1 [file plants-15-03174-s001.zip › Table S3.pdf]

Table S3. The sequences of PLCPs from *P. trichocarpa*, *V. vinifera* and *A. thaliana*

>PtrRD21C
MASLHRSLAFMAMLSLLSVCLAIDMSIIDYNIKHGQVPERTEAETRRIYEMWLVKHGRAYNALGEKERRFEIFKDNLKFI
DEHNSVGNPSYKLGLNKFADLSNDEYRSVYLGTRMDGKGRLLGGPKSERYLFKEGDDLPETVDWREKGAVAPVKDQG
QCGSCWAFSTVGAVEGINQIVTGNLTSLSEQELVDCDKTYNLGCNGGLMDYAFDFIIENGGIDTEEDYPYKAIDSMCDPN
RKNARVVTIDGYEDVPQNDEKSLKKAVANQPVSVAIEAGGRGFQLYQSGVFTGSCGTQLDHGVVTVGYGTEHGVDYWI
VRNSWGPAWGENGYIRMERDVASTETGKCGIAMEASYPTKKSANPPNPGPSPPSPVNPPPPEKPSSECDDYYSCPAGSTC
CCIYQYGDYCFGWGCCPLESATCCDDHYSCCPHEYPVCDLEAGTCRMSKSNPFGVKMLTRAPARITQSHQLGGRRRAGR
RFSPA*

>PtrXCP2A
MALSSLSLMFLLAFSFMSFFANSGLARDFSIVGYTPEDLTSGDKIIDLFESWISKHGKIYESMEEKWLRFEIFKDNLFHIDET
NKKVVNYWLGLNEFSDLSHEEFKNKYLGLKVDMSERRECSQEFNYKDVMSIPKSVDWRKKGAVTDVKNQGSCGSCWA
FSTVAAVEGINQIVTGNLTSLSEQELVDCDTTNNYGCNGGLMDYAFSYIISNGGLHKEVDYPYIMEEGTCEMRKEESEVV
TISGYHDVPQNSEESLLKALANQPLSVAIEASGRDFQFYSGGVFDGHCGTQLDHGVAAVGYGSTNGLDYIIVKNSWGSK
WGEKGYIRMKRNTGKPAGLCGINKMASYPTKKK*

>PtrRD19C
MERFSLLALLLLTLICSAVASTVSSNDLDDPLIRQVVSDGEDDLLNAEHHFTSFKSKFGKTYATQEEHDYRFGVFKANLRR
AKKHQMIDPTAAHGITKFSDLTPKEFRRQFLGLKRWLRLPTDANKAPILPTTDLPTDYDWRDHGAVTEVKDQGSCGSCW
SFSATGALEGAHYLATGELASLSEQQLVDCDHECDPEEYGACDSGCDGGLMNNAFEYALKAGGLEREEDYPYTGTDGG
TCKFDKSKVVASVSNFSVVSIDEDQIAANLVKHGPLSVAINAAFMQTYVGGVSCPYICSKRQDHGVLLVGYGSAGYAPIR
FKEKPFWIIKNSWGQNWGENGYYKICRGRNICGVDSMVSTVAAIHTTAQ*

>PtrSAG12A
MAAKKCSIHIFLPFLLILATKIVCRPLDEQEYMLKRHEEWMAQHGRVYGDMKEKEKRYLIFKENIERIEAFNNGSDRGYK
LGVNKFADLTNEEFRAMHHGYKRQSSKLMSSSFRHENLSAIPTSMDWRKAGAVTPVKDQGTCGCCWAFSAVAAIEGIIK
LKTGKLISLSEQQLVDCDVKGVDQGCGGGLMDNAFQFILRNGGLTSEATYPYQGVDGTCKSKKTASIEAKITGYEDVPV
NNENALLQAVAKQPVSVAVEGGGYDFQFYKSGVFKGDCGTYLDHAVTAIGYGTNSDGTNYWLVKNSWGTSWGESGYM
RMQRGIGAREGLCGVAMDASYPTA*

>PtrSAG12E
MAAKKCNTRIFVPFLLILAAWATKIACRPLDEQEYMLKRHEEWMAQHGRVYGDMKEKEKRYLIFKENIERIEAFNNGSD
RGYKLGVNKFADLTNEEFRAMYHGYKRQSSKLMSSSFRYENLSDIPTSMDWRNDGAVTPVKDQGTCGCCWAFSTVAAI
EGIIKLQTGNLISLSEQQLVDCTAGNKGCQGGHMDTAFQYIIRNGGLTSEDNYPYQGVDGTCSSEKAASTEAQITGYEDVP
QNNENALLQAVAKQPVSVAVEGGGHDFQFYKSGVFEGDCGTNLNHAVTAIGYGTDSDGTDYWLVKNSWGTSWGENGY
MRMRRGIGSSEGLCGVAKDASYPTA*

>PtrSAG12B
MVGCCWAFSAVAAIEGIIKLKTGNLISLSEQQLVNRDVGNKGCHGGLMDTAFQYIIRNEGLTSEDNYPYQGVDGTCSSEK
ATSIAAEITGDENVPKNNENALLQAVAKQPVSVGVDGGGNDFQFYKSGVFNGDCGTQQNHAVTAIGYGTDSDGTDYWL
VKNSWGTSWGESGYTRMQRGIGASEGLYGVAMDASYPTA*

>PtrSAG12H
MAAKKCNTRIFLPFLLILAAWATKIACRPLDEQEYMLKRHEEWMAQHGRVYGDMKEKEKRYLIFKENIERIEAFNNGSD
RGYKLGVNKFADLTNEEFRAMYHGYKRQSSKLMSSSFRYENLSDIPTSMDWRNDGAVTPVKDQGTCGCCWAFSTVAAI
EGIIKLQTGNLISLSEQQLVDCTAGNKGCQGGLMDTAFQYIIRNGGLTSEDNYPYQGVDGTCSSEKAASTEAQITGYEDVP
QNNENALLQAVAKQPVSVAVDGGGNDFRFYKSGVFEGDCGTNLNHGVTAIGYGTDSDGTDYWLVKNSWGTSWGENGY
MRMRRGIDSSEGLCGVAMDASYPTA*

>PtrSAG12D
MAAKKCNTRIFVPFLLILAAWATKIACRPLDEQEYMLKRHEEWMAQHGRVYGDMKEKEKRYLIFKENIERIEAFNNGSD
RGYKLGVNKFADLTNEEFRAMYHGYKRQSSKLMSSSFRYENLSDIPTSMDWRNDGAVTPVKDQGTCGCCWAFSTVAAI
EGIIKLQTGNLISLSEQQLVDCTAGNKGCQGGLMDTAFQYIIRNGGLTSEDNYPYQGVDGTCSSEKAASTEAQITGYEDVP
QNNENALLQAVAKQPVSVGVDGGGNDFQFYKSGVFNGDCGTQQNHAVTAIGYGTDIDGTDYWLVKNSWGTSWGENG
YMRMRRGIGSSEGLCGVAMAASYPTA*

>PtrSAG12G
MAAKKCNTRIFVPFLLILAAWATKIACRPLDEQEYMLKRHEEWMAQHGRVYGDMKEKEKRYLIFKENIERIEAFNNGSD
RGYKLGVNKFADLTNEEFRAMYHGYKRQSSKLMSSSFRYENLSDIPTSMDWRNDGAVTPVKDQGTCGCCWAFSTVAAI
EGIIKLQTGNLISLSEQQLVDCTAGNKGCQGGLMDTAFQYIIRNGGLTSEDNYPYQGVDGTCSSEKAASTEAQITGYEDVP
QNNENALLQAVAKQPVSVAVDGGGNDFRFYKSGVFEGDCGTNLNHGVTAIGYGTDSDGTDYWLVKNSWGTSWGENGY
MRMRRGIGSSEGLCGVAMDASYPTA*
>PtrSAG12C
MVGCCWAFSAVAAIEGIIKLKTGNLISLSKQQLVNRDVGNKGCHGGLMDTAFQYIIRNEGLTSEDNYPYQGVDGTCSSEK
AASIAAEITGDENAPKNNENALLQAVAKQPVSVGVDGGGNDFQFYKSGVFNGDCGTQQNHAVTAIGYGTDSDGTDYWL
VKNSWGTSWGESGYTRMQRGIGASEGLCGVAMDASYPTA*
>PtrSAG12I
MAAKKCNTRIFLPFLLILAAWATKIACRPLDEQEYMLKRHEEWMAQHGRVYGDMKEKEKRYLIFKENIERIEAFNNGSD
RGYKLGVNKFADLTNEEFRAMYHGYKRQSSKLMSSSFRYENLSDIPTSMDWRNDGAVTPVKDQGTCGCCWAFSTVAAI
EGIIKLQTGNLISLSEQQLVDCTAGNKGCQGGLMDTAFQYIIRNGGLTSEDNYPYQGVDGTCSSEKAASTEAQITGYEDVP
QNNENALLQAVAKQPVSVAVDGGGNDFRFYKSGVFEGDCGTNLNHGVTAIGYGTDSDGTDYWLVKNSWGTSWGENGY
MRMRRGIGSSEGLCGVAMDASYPTA*
>PtrSAG12F
MAAKKCNTRIFVPFLLILAAWATKIACRPLDEQEYMLKRHEEWMAQHGRVYGDMKEKEKRYLIFKENIERIEAFNNGSD
RGYKLGVNKFADLTNEEFRAMYHGYKRQSSKLMSSSFRYENLSDIPTSMDWRNDGAVTPVKDQGTCGCCWAFSTVAAI
EGIIKLQTGNLISLSEQQLVDCTAGNKGCQGGLMDTAFQYIIRNGGLTSEDNYPYQGVDGTCSSDKAASTEAQITGYEDVP
QNNENALLQAVAKQPVSVAVDGGGNDFRFYKSGVFEGDCGTNLNHAVTAIGYGTDSDGTDYWLVKNSWGTSWGENGY
MRMRRGIGSSEGLCGVAKDASYPTA*
>PtrXBCP3B
MSSEMEDFKKSQMVHLILLLLVGLTSVSSSLPSEYSIVGNDFSELPPDESIIEIFQQWRDRHQKAYKHAEEAEKRFGNFKR
NLKYIIEKTGKETTLRHRVGLNKFADLSNEEFKQLYLSKVKKPINKTRIDAEDRSRRNLQSCDAPSSLDWRKKGVVTAVK
DQGDCGSCWSFSTTGAIEGINAIVTSDLISLSEQELVDCDTTNYGCERGYMDYAFEWVINNGGIDTEANYPYTGVDGTCN
TAKEEIKVVSIDGYKDVDETDSALLCAAAQQPISVGIDGSAIDFQLYTGGIYDGDCSDDPDDIDHAVLIVGYGSENGEDY
WIVKNSWGTSWGIEGYFYIKRNTDLPYGVCAINAMASYPTKEASAQSPTSPPSPPSPPPPPPPPPTPVPPPPSPQPSDCGDFS
YCPSDETCCCILNVFDYCLVYGCCAYENAVCCADSVYCCPSDYPICDVEEGLCLKGQGDYLGVAASKRHMAKHKFPWT
KLQERAKTDHRVLQWKRNPFAAMR*
>PtrRD19B
MSLNLSLLVILSLLFISAVHAETLNGDDPLIREVVDGQDASSSNLLSAEQHHFSLFKSKFKKSYGSQEEHDYRFSVFKANL
RRAARHQELDPTASHGVTQFSDLTPAEFRKQVLGLRRLRLPKDANEAPILPTSDLPEDFDWRDKGAVGPIKNQGSCGSCW
SFSATGALEGAHFLATGELVSLSEQQLVDCDHECDPEEPGSCDSGCNGGLMNSAFEYTLKAGGLMREEDYPYTGTDRDA
CKFDKNKVAARVANFSVVSLDEDQIAANLVKNGPLAVAINAVFMQTYIGGVSCPYICSRRLDHGVLLVGYGSAGYSPVR
MKEKPFWIIKNSWGEKWGENGFYKICRGRNVCGVDSMVSTVAAVQTSSQ*
>PtrXCP1
MALSVLKTSFLTFFASLFVCSVLAHDFSIVGYSPEHLTSVDKLVELFESWISGHGKAYNSLEEKLHRFEVFKENLKHIDQR
NKEVTSYWLGLNEFADLSHEEFKSKFLGLYPEFPRKKSSDDFSYRDVVDLPKSIDWRKKGAVTPVKNQGSCGSCWAFST
VAAVEGINQIVAGNLTSLSEQQLIDCDTSFNNGCNGGLMDYAFEFIVNNGGLHKEEDYPYLMEEGTCDEKREEMEVVTIS
GYHDVPRNDEQSLLKALAHQPLSVAIDASGRDFQFYSGGVFSGPCGTDLDHGVAAVGYGSSSGIDYIIVKNSWGPKWGE
RGYLRMKRNTGKPEGLCGINKMASYPTKQK*
>PtrSAG12O
MQRDMCACLLSNSFTTIVAATTAKSIMRLTKQSQFICLALLFVLGAWPSKSAARTLQDVSMYERHEQWMAQYGRVYKD
DAEKETRYNIFKENVARIDAFNSQTGKSYKLGVNQFADLSNEEFKASRNRFKGHMCSPQAGPFRYENVSAVPATMDWRK
KGAVTPVKDQGQCGCCWAFSAVAAMEGINQLTTGKLISLSEQEVVDCDTKGEDQGCNGGLMDDAFKFIEQNKGLTTEA
NYPYTGTDGTCNTQKEATHAAKITGFEDVPANSEAALMKAVAKQPVSVAIDAGGFEFQFYSSGIFTGSCGTQLDHGVTAV
GYGISDGTKYWLVKNSWGAQWGEEGYIRMQKDISAKEGLCGIAMQASYPSA*
>PtrSAG12N

MKFTKQSQFICLALLFVLGAWPSKSVARTLQDVSMYERHEQWMAQYGRVYKDDAEKETRYNIFKENVARIDAFNSQTG
KSYNLGVNQFADLTNEEFKASRNRFKGHMCSPQAGPFRYENVSAVPATMDWRKKGAVTPVKDQGQCGCCWAFSAVAA
MEGINQLTTGKLISLSEQEVVDCDTKGEDQGCNGGLMDDAFKFIEQNKGLTTEANYPYTGTDGTCNTQKEVSHAAKITG
FQDVPANSEAALMKAVAKQPVSVAIDAGGFEFQFYSSGIFTGSCGTELDHGVTAVGYGGSDGTKYWLVKNSWGAQWGE
EGYIRMQKDISAKEGLCGIAMQASYPTA*
>PtrRDL1B
MALAISALLFLLFTMSSALDMSILTSNDNQLNHDRSSWRSDDEVMSMYKWWLAKHGKAYNGLGEEAERFEIFKNNLRFI
DEHNSQNHTYKVGLTKFADLTNEEYRAMFLGTRSDAKRRLMKSKSPSERYAFKAGDKLPESVDWRAKGAVNPIKDQGS
CGSCWAFSTVAAVEGINQIVTGELISLSEQELVDCDRTYNAGCNGGLMDYAFQFIINNGGLDTEKDYPYVGDDDKCDKD
KMKTKAVSIDGFEDVLPYDEKALQKAVAHQPVSVAIEASGMALQFYQSGVFTGECGTALDHGVVVVGYASENGLDYWL
VRNSWGTEWGEHGYIKMQRNVGDTYTGRCGIAMESSYPVKNGENTAKPNLAEAKQEGKVIEGVKLDDW*
>PtrXBCP3A
MNFLYIFALTLLISVLSPSTSSSDISQLFETWCKEHGKSYTSQEERSHRLKVFEDNYDFVTKHNSKGNSSYSLALNAFADLT
HHEFKTSRLGLSAAPLNLAHRNLEITGVVGDIPASIDWRNKGVVTNVKDQGSCGACWSFSATGAIEGINKIVTGSLVSLSE
QELIECDKSYNDGCGGGLMDYAFQFVINNHGIDTEEDYPYRARDGTCNKDRMKRRVVTIDKYVDVPENNEKQLLQAVA
AQPVSVGICGSERAFQMYSKGIFTGPCSTSLDHAVLIVGYGSENGVDYWIVKNSWGTGWGMRGYMHMQRNSGNSQGV
CGINMLASYPVKTSPNPPPPPPPGPTKCNLLTYCAAGETCCCARKFFGICISWKCCGLDSAVCCKDRLHCCPHDYPVCDT
DKNMCFKRAGNATRMEAIEGKTSGKFGSWNSLPEAWIQ*
>PtrRD19E
MERLPLLFLLLLTALSSTLASTVSSSDLDDPLIRQVVSEGEDHLLNAEHHFTTFKSKFGKNYATQEEHDYRFSVFKANLLR
AKKHQIMDPTAAHGVTKFSDLTPKEFRRQLLGLKRRLRLPTDANKAPILPTGDLPTDFDWRDHGAVTSVKDQGSCGSCW
SFSATGALEGAHYLATGELVSLSEQQLVDCDHECDPEEYGACDSGCSGGLMNNAFEYALKAGGLEREKDYPYTGNDRG
ACKFEKSKVAASVSNFSVVSLDEDQIAANLVKHGPLSVAINAVFMQTYIGGVSCPYICSKHQDHGVLLVGYGAAGYAPIR
FKEKPFWIIKNSWGENWGENGYYKICRARNICGVDSMVSTVAAIHATAQ*
>PtrXCP2B
MALPALSLILALSLMSFFASSCLARDFSIVGYAPEDLTSRDRIIDLFESWISKHQKIYESIEEKWHRFEIFKDNLFHIDETNKK
VVNYWLGLNEFADLSHEEFKNKYLGLNVDLSNRRECSEEFTYKDVSSIPKSVDWRKKGAVTDVKNQGSCGSCWAFSTV
AAVEGINQIVTGNLTSLSEQELVDCDTTYNNGCNGGLMDYAFAYIISNGGLHKEEDYPYIMEEGTCEMRKAESEVVTISG
YHDVPQNSEESLLKALANQPLSVAIDASGRDFQFYSGGVFDGHCGTELDHGVAAVGYGSAKGLDFIVVKNSWGSKWGE
KGFIRMKRNTGKPAGLCGINKMASYPTKKK*
>PtrALP2
MARVAGLVVSSILFLLCCVAAGSSFDESNPIKLVSDRLHDFESSFVKVLGQSRRALSFARFAHRHGKRYETEGEMKLRFAIF
SESLDLIRSTNKKGLPYTLGLNQFADWTWQEFQKYRLGAAQNCSATTRGNHKLTNALLPETKDWREEGIVSPVKNQGH
CGSCWTFSTTGALEAAYHQAFGKGISLSEQQLVDCARAFNNFGCNGGLPSQAFEYIKFNGGLDTEEAYPYTGKDDACKF
SSENVGVRVVESVNITLGAEDELKHAVAFVRPVSVAFEVVGSFRLYKEGVYTTSTCGSTPMDVNHAVLAVGYGVENGIPY
WLIKNSWGEDWGDNGYFKMEMGKNMCGIATCASYPVVAA*
>PtrRDL1A
MALAISTLLFLFFTVSSALDMSIFNHDDNHLSHDQSSWRSDDEVMSIYKWWLQKHGKAYNRLGEKAKRFEIFKNNLRFI
DEHNSQNRTYKVGLTKFADLTNQEYRAMFLGTRSDPKRRLMKSKNPSERYAYKAGDKLPESVDWRGKGAVNPIKDQGS
CGSCWAFSTVAAVEGINQIVTGELISLSEQELVDCDRFYNAGCNGGLMDYAFQFIINNGGLDTEKDYPYLGNDDTCDRDK
MKTKAVSIDGFEDVLPFDEKALQKAVAHQPVSVAIEASGMALQFYQSGVFTGECGTALDHGVVVVGYGTEKGLDYWLV
RNSWGTEWGEHGYIKMQRNVRDTYTGRCGIAMESSYPVKNGQNTAKPYLADESAGEKISSI*
>PtrSAG12R
MPTSLLIASLIYLLLIPKAAMRFTKQFQFGCLALLFILGAWPSKSTARTLLDAPMYERHEQWMTQYGRVYKDDTERATRY
SIFKENVARIDAFNSQTGKSYKLGVNQFADLTNEEFKASRNRFKGHMCSPQAGPFRYENVSAVPSTMDWRKEGAVTPVK
DQGQCGCCWAFSAVAAMEGINKLTTGKLISLSEQEVVDCDTKGEDQGCNGGLMDDAFKFIEQNKGLTTEANYPYKGTD
GTCNTNKAAIHAAKITGFEDVPANSEAALMKAVAKQPVSVAIDAGGSDFQFYSSGIFTGSCDTQLDHGVTAVGYGVSDGS
KYWLVKNSWGAQWGEEGYIRMQKDISAKEGLCGIAMQASYPTA*
>PtrSAG12Q

MPTSLLIASPIYQLLIPKAAMRFTKQFQFVCLALLFILGAWPSKSTARTLLDAPMYERHEQWMTQYGRVYKDDNERATRY
SIFKENVARIDAFNSQTGKSYKLGVNQFADLTNEEFKASRNRFKGHMCSPQAGPFRYENVSAVPSTVDWRKEGAVTPVK
DQGQCGCCWAFSAVAAMEGINKLTTGKLISLSEQEVVDCDTKGEDQGCNGGLMDDAFKFIEQNKGLTTEANYPYKGTD
GTCNTNKAAIHAAKITGFEDVPANSEAALMKAVAKQPVSVAIDAGGSDFQFYSSGIFTGSCDTQLDHGVTAVGYGVSDGS
KYWLVKNSWGAQWGEEGYIRMQKDISAKEGLCGIAMQASYPTA*
>PtrSAG12P
MRFTKQFQFVCLALLFILGAWPSKSTARTLLDAPMYERHEQWMTQYGRVYKDDNERATRYSIFKENVARIDAFNSQTGK
SYKLGVNQFADLTNEEFKASRNRFKDHMCSPQAGPFRYENVSAVPSTVDWRKEGAVTPVKDQGQCGCCWAFSAVAAME
GINKLTTGKLISLSEQEVVDCDTKGEDQGCNGGLMDDAFKFIEQNKGLTTEANYPYKGTDGTCNTNKAAIHAAKITGFE
DVPANSEAALMKAVAKQPVSVAIDAGGSDFQFYSSGIFTGSCDTQLDHGVTAVGYGVSDGSKYWLVKNSWGAQWGEEG
YIRMQKDISAKEGLCGIAMQASYPTA*
>PtrCEP1A
MDTRKVILAVFSVVLVFRLADSFDYTEEDLASEERLRDLYERWRSHHTVSRSLAEKQERFNVFKENLKHIHKVNHKDRP
YKLKLNSFADMTNHEFLQHYGGSKVSHYRVLRGQRQGTGSMHEDTSKLPSSVDWRKNGAVTGIKDQGKCGSCWAFST
VAAVEGINKIKTGELISLSEQELVDCDSDNHGCNGGLMEDAFNFIKQIGGLTSENTYPYRAKEEPCDSNKMNSPVVNIDGY
EMVPENDENALMKAVANQPVAIAMDAGGKDLQFYSEGVFTGDCGTELNHGVALVGYGTTQDGTKYWIVKNSWGTDW
GEKGYIRMQRGIDAEEGLCGITMEASYPVKLRSDNKKAPSRKDEL*
>PtrRD21B
MASLYRSFAFLATFYFLSVCLAIDMSIIDYNLKHGQVPERTEAETLRLYEMWLVKYGKAYNALGEKERRFEIFKDNLKFV
DQHNSVGNPSYKLGLNKFADLSNEEYRAAYLGTRMDGKRRLLGGPKSARYLFKDCDDLPESVDWREKGAVAPVKDQG
QCGSCWAFSTVGAVEGINQIVTGNLTSLSEQELVDCDKVYNQGCNGGLMDYAFEFIMKNGGIDTEEDYPYKAVDSMCDP
NRKNARVVTIDGYEDVPQNDEKSLRKAVANQPVSVAIEAGGRAFQLYQSGVFTGSCGTQLDHGVVVVGYGTENGVDY
WVVRNSWGPAWGENGYIRMERNVASTETGKCGIAMEASYPTKKGANPPNPGPSPPSPVNPSPPPSSECDDYYSCPAGSTC
CCIYPYGDYCFGWGCCPLESATCCDDHNSCCPHEYPVCDLEAGTCRMSKNNPFGVKALTRAPARIAQSHQLGGKRPAGR
RLGIV*
>PtrRD19A
MSLNLSLFLILSLFFISAISAETFNGDDSLIRQVVEGQDESSSNLLTAEQHHFSLFKRKFKKSYLSQEEHDYRFSVFKSNLRR
AARHQKLDPTASHGVTQFSDLTSAEFRKQVLGLRKLRLPKDANTAPILPTNDLPEDFDWREKGAVGPVKNQGSCGSCWS
FSTTGALEGAHFLATGELVSLSEQQLVDCDHECDPEEPGSCDSGCNGGLMNSAFEYTLKAGGLMREEDYPYTGMDRGA
CKFDKNKVAAGVANFSVVSLDEDQIAANLVKNGPLAVAINAVFMQTYIGGVSCPYICSRRLDHGVLLVGYGSAAYAPVR
MKEKPYWIIKNSWGESWGENGFYKICRGRNICGVDSMVSTVAAVQTNSL*
>PtrRD19D
MIHKTPALTCTAILTVLLAFSSVSVAVTDLYDNPQDPTILQVTDNPTLDNRKFMKNGLNLLGTEEKFKMFIKEHNKEYATR
EEYVHRFGIFGKNLIRAVEHQALDPTAIHGVTPFMDLTEEEFERMYAGVLGGGTVPVEKGSVSFMDASGLPDSFDWREK
GAVTDVKIQGSCGSCWAFSTTGSVEGANFIATGKLLNLSEQQLVDCDRVCDKTDKASCDDGCGGGLMTNAYRYLIEAGG
LQEESSYPYTGKSGECKFDPEKIAVKVANFTSIAVDENQIAANLVHHGPLAIGLNAIFMQTYIGGVSCPLICGKKWLNHGV
LLVGYGARGYSILRFGYKPYWIIKNSWGNHWGEKGYYRLCRGHGMCGMNKMVSAVVTKVA*
>PtrSAG12J
MAFIPENKLIFAVVLLLGLWTSHVWSRSLYEASMMERHETWMAQYGRAYKGHVEKERRLNIFKNNVEFIESFNKVGKKP
YKLSVNEFADLTNEEFQASRNGYKMSAHLSSSSTKPFRYENVSAVPSTMDWRKKGAVTPIKDQGQCGCCWAFSAVAATE
GITQLSTGKLISLSEQELVDCDTSGEDQGCNGGLMDDAFDFIIQNKGLTTEANYPYQGADGACNSGKAAAKITGYEDVPA
NSEAALLKAVANQPVSVAIDAGGSAFQFYSSGVFTGDCGTDLDHGVTAVGYGMSDDGTKYWLVKNSWGTSWGENGYI
RMERDIDAQEGLCGIAMEASYPTA*
>PtrXBCP3C
MDSKKSQMSLIIFLLLALLTCLSSSLPGEHPIVVNDFSELVSEESIIEIFQQWRDRHQKVYEHAAESEKRYRNFKRNLKYIIE
KAGKKTAALGHSVGLNKFADLSNEEFKELYLSKVKKPINIKRSTARDWRQRNLQTCDAPSSLDWRKKGVVTAVKDQGD
CGSCWSFSTTGAIEGINAIVTGDLISLSEQELVDCDTTNYGCEGGYMDYAFEWVINNGGIDTEANYPYTGVDGTCNTTKE
EIKVVSIDGYTDVDETDSALLCATVQQPISVGMDGSALDFQLYTGGIYDGDCSDDPNDIDHAVLIVGYGSENGEDYWIVK
NSWGTEWGMEGYFYIKRNTDLPYGVCAINAEASYPTKESSSPSPTSPPSPPSPLSPPPPPPPPTPVPPPPCPQPSDCGDFAYC

PSDETCCCILKVFDYCIVYGCCQYENAVCCADSVYCCPSDYPICDVEEGLCLKSQGDYLGVPASKRHMAKHKFPWTKLE
EKTTTDRHALRWKRNPFDAMR*
>PtrXBCP3D
MESQKQQLTLFIFLLLAPLACLSSGLPGEYSTVSNDLHEGLTEEGITEVFKLWKEKHQKVYKHAEEAERRIGNFKRNLKYI
IEKNGKRKSGLEHKVGLNKFADLSNEEFREMYLSKVKKPITIEEKRKHRHLQTCDAPSSLDWRNKGVVTAVKDQGDCGS
CWSFSTTGAIEAINAIVTGDLISLSEQELVDCDTTNNYGCEGGDMDSAFQWVIGNGGIDTEADYPYTGVDGTCNTAKEEK
KVVSIEGYVDVDPSDSALLCATVQQPISVGMDGSALDFQLYTGGIYDGDCSGDPNDIDHAILIVGYGSENDEDYWIVKNS
WGTEWGMEGYFYIRRNTSKPYGVCAINADASYPTKVPSPPSPPSPPPPPSPPPPPPSPPPPCPQPSDCGDSSFCPSDETCCCI
LKLFSSCIIYGCCPYENAVCCAESTYCCPSDYPICDVDDGLCLRGQGDHLGVAARRRHMANYKFPWTKFEEIKETKQPVL
QWKRSLRCNALNYME*
>PtrCEP1B
MEMKKFLPVALSLALVLGITESLGFHGKDLESEESLWDLYERWRSHHTVSRSLDEKHKRFNVFKENVIHVHKTNKMDKP
YKLKLNKFADMTNHEFRSVYAGSKVKRHKMFCGTTPRGNGSFMYEKVDEVPSSVDWRKKGAVTAVKDQGQCGSCWA
FSTIVAVEGINYIKTNKLVSLSEQELVDCDTTENQGCNGGLMEYAFEFIKKKGGVTTESTYPYQAEDGTCDVEKVNKPVV
SIDGYEKVPENDEDALLKAAANQPISVAIDAGGSDFQFYSEGVFTGKCGTELDHGVAIVGYGTTLDGTKYWVVRNSWGP
EWGEKGYIRMQRGISDKEGLCGIAMEASFPIKNSSTNPSGTRSSPKDEL*
>PtrSAG12L
MSARHEQWMETYGKVYADAAEKERRFEIFKDNVEYIESFNTAGNKPYKLSVNKFADLTNEELKVARNGYRRPLQTRPM
KVTSFKYENVTAVPATMDWRKKGAVTPIKDQGQCGSCWAFSTVAATEGINQLTTGKLVSLSEQELVDCDTQGEDQGCEG
GLMEDGFEFIIKNHGITTEANYPYQAADGTCNSRKEASRIAKITGYESVPANSEAALLKAVASQPISVSIDAGGSDFQFYSS
GVFTGQCGTELDHGVTAVGYGETSDGTKYWLVKNSWGTSWGEEGYIRMQRDTEAEEGLCGIAMDSSYPTA*
>PtrSAG12K
MVSICRRLCFFAFILILGMWAYEVASRELQEPSMSARHEQWMATFGKVYADAAETERRFEIFKDNVEYIESFNTTGNKPY
KLSVNKFADQTNEELKVARNGYRRPLQTRPMKVTSFKYENVTAVPATMDWRKKGAVTLIKDQGQCGSCWAFSTVAATE
GINQLTTGKLVSLSEQELVDCDIQGEDQGCEGGLMEDGFEFIIKNHGITTEANYPYQAADGTCNSKMEASRIAKITGYESV
PANSEAALLKAVANQPISVSIDAGGSDFQFYSSGDFTGQCGTELDHGVTAVGYGEISDGTKYWLVKNSWGTSWGEEGYIR
MQRDTEAEEGLCGIAMDSSYPTA*
>PtrSAG12M
MVSICKRQCFFAFILILGMWAFEVASRELQESYMSARHEQWMATYGKVYVDAAEKERRFKIFKNNVEYIESFNTAGNKP
YKLSVNKFADQTNEKFKGARNGYRRPFQTRPMKVTSFKYENVTAVPATMDWRKKGAVTPIKDQGQCGSCWAFSTVAAT
EGINQLTTGKLVSLSEQELVDCDNQGEDQGCEGGLMEDGFEFIIKNHGITTEANYPYQAADGTCNSKKQASHIAKITGYE
SVPANSEAELLKVVANQPISVSIDAGGSDFQFYSSGVFTGKCGTELDHGVTAVGYGETSDGTKYWLVKNSWGTSWGEEG
YIRMQRDIDTEEGLCGIAMDSSYPTA*
>PtrRD21A
MGLFRSSTLMLMLLFLVFALSSAFDMSIISYHQTHATKSSWRTDDEVMAMYEEWLVKHGKNYNALGEKEKRFEIFKDNL
MFIDQHNSENRTYTVGLNRFADLTNEEFRSMYLGTRTGHKKRLPKTSDRYAPRVGDSLPDSVDWRKEGAVAEVKDQGGC
GSCWAFSTIAAVEGINKIVTGDLIALSEQELVDCDTSYNEGCNGGLMDYAFEFIINNGGIDTEDDYPYLGRDGRCDTYRKN
AKVVSIDSYEDVPENDETALKKAVANQPVSVAIEGGGRNFQLYNSGVFTGECGTSLDHGVAAVGYGTEKGKDYWIVRNS
WGKSWGESGYIRMERNIASPTGKCGIAIEPSYPIKKGQNPPNPGPSPPSPVKPPSVCDNYFSCPDSSTCCCIFEYGKYCFAW
GCCPLEGATCCDDHYSCCPHEYPVCNVNEGTCLISKGNPFGVKALRRTPAKPHWAHGTEGKNSVA*
>PtrCEP1C
MEMKKFLFVALSLALVLGITESLDFHEKDLESEESLWDLYERWRSHHTVSTSLDEKHKRFNVFKENVMHVHKTNKMGK
PYKLKLNKFADMTNHEFRSVYAGSKVKHHRMFRGTTRGNGSFMYGKVEKVPTSVDWRKKGAVTAVKDQGQCGSCWA
FSTIVAVEGINYIKTNELVSLSEQELVDCDTTENQGCNGGLMEYAFEFIKKKRGITTESTYPYKAEDGHCDAAKENNPAVSI
DGYEKVPENDEDALLKAAANQPVSVAIDAGGSDFQFYSEGVFIGECGTELDHGVAVVGYGTTLDGTKYWIVRNSWGPE
WGEKGYIRMQRGISDKEGLCGIAMEASYPIKNSSTNPSGTKSSPKDEL*
>PtrCEP1D
MKKLLFVALYLALVLGFTESFDFHEKDLESEESLWDLYEKWRSHHTVSTSLDEKRKRFNVFRANVLHVHNTNKMDKPY
KLKLNKFADMTNHEFRTAYASSKVKHHTMFRGAPLGNGSFMYGNIDKVPASIDWRKKGAVTPVKDQGKCGSCWAFSTI

VAVEGINFIKTNKLISLSEQELVDCNTGENHGCNGGLMDYAFEFITKQKGITTEANYPYRAQDGHCDANKANQPAVSIDG
HEDVLHNNENALLKAVANQPVSVAIDAGGSDFQFYSEGVFTGECGKELDHGVAIVGYGTTVDGTKYWIVRNSWGPEWG
ERGYIRMQRGISDRRGLCGIAMEASYPIKKSSTNPIGPADSPKDEL*
>AtCEP1
MKRFIVLALCMLMVLETTKGLDFHNKDVESENSLWELYERWRSHHTVARSLEEKAKRFNVFKHNVKHIHETNKKDKSY
KLKLNKFGDMTSEEFRRTYAGSNIKHHRMFQGEKKATKSFMYANVNTLPTSVDWRKNGAVTPVKNQGQCGSCWAFSTV
VAVEGINQIRTKKLTSLSEQELVDCDTNQNQGCNGGLMDLAFEFIKEKGGLTSELVYPYKASDETCDTNKENAPVVSIDG
HEDVPKNSEDDLMKAVANQPVSVAIDAGGSDFQFYSEGVFTGRCGTELNHGVAVVGYGTTIDGTKYWIVKNSWGEEWG
EKGYIRMQRGIRHKEGLCGIAMEASYPLKNSNTNPSRLSLDSLKDEL*
>AtCEP2
MKLFFIVLISFLSLLQASKGFDFDEKELETEENVWKLYERWRGHHSVSRASHEAIKRFNVFRHNVLHVHRTNKKNKPYK
LKINRFADITHHEFRSSYAGSNVKHHRMLRGPKRGSGGFMYENVTRVPSSVDWREKGAVTEVKNQQDCGSCWAFSTVA
AVEGINKIRTNKLVSLSEQELVDCDTEENQGCAGGLMEPAFEFIKNNGGIKTEETYPYDSSDVQFCRANSIGGETVTIDGHE
HVPENDEEELLKAVAHQPVSVAIDAGSSDFQLYSEGVFIGECGTQLNHGVVIVGYGETKNGTKYWIVRNSWGPEWGEGG
YVRIERGISENEGRCGIAMEASYPTKLSSTPSTHESVVRDDVKDEL*
>AtCEP3
MKKLLLIFLFSLVILQTACGFDYDDKEIESEEGLSTLYDRWRSHHSVPRSLNEREKRFNVFRHNVMHVHNTNKKNRSYKL
KLNKFADLTINEFKNAYTGSNIKHHRMLQGPKRGSKQFMYDHENLSKLPSSVDWRKKGAVTEIKNQGKCGSCWAFSTVA
AVEGINKIKTNKLVSLSEQELVDCDTKQNEGCNGGLMEIAFEFIKKNGGITTEDSYPYEGIDGKCDASKDNGVLVTIDGHE
DVPENDENALLKAVANQPVSVAIDAGSSDFQFYSEGVFTGSCGTELNHGVAAVGYGSERGKKYWIVRNSWGAEWGEGG
YIKIEREIDEPEGRCGIAMEASYPIKLSSSNPTPKDGDVKDEL*
>AtRD21A
MGFLKPTMAILFLAMVAVSSAVDMSIISYDEKHGVSTTGGRSEAEVMSIYEAWLVKHGKAQSQNSLVEKDRRFEIFKDNL
RFVDEHNEKNLSYRLGLTRFADLTNDEYRSKYLGAKMEKKGERRTSLRYEARVGDELPESIDWRKKGAVAEVKDQGGC
GSCWAFSTIGAVEGINQIVTGDLITLSEQELVDCDTSYNEGCNGGLMDYAFEFIIKNGGIDTDKDYPYKGVDGTCDQIRKN
AKVVTIDSYEDVPTYSEESLKKAVAHQPISIAIEAGGRAFQLYDSGIFDGSCGTQLDHGVVAVGYGTENGKDYWIVRNSW
GKSWGESGYLRMARNIASSSGKCGIAIEPSYPIKNGENPPNPGPSPPSPIKPPTQCDSYYTCPESNTCCCLFEYGKYCFAWG
CCPLEAATCCDDNYSCCPHEYPVCDLDQGTCLLSKNSPFSVKALKRKPATPFWSQGRKNIA*
>AtRD21B
MGFLKLSPMILLLLAMIGVSYAMDMSIISYDENHHITTETSRSDSEVERIYEAWMVEHGKKKMNQNGLGAEKDQRFEIFK
DNLRFIDEHNTKNLSYKLGLTRFADLTNEEYRSMYLGAKPTKRVLKTSDRYQARVGDALPDSVDWRKEGAVADVKDQG
SCGSCWAFSTIGAVEGINKIVTGDLISLSEQELVDCDTSYNQGCNGGLMDYAFEFIIKNGGIDTEADYPYKAADGRCDQNR
KNAKVVTIDSYEDVPENSEASLKKALAHQPISVAIEAGGRAFQLYSSGVFDGLCGTELDHGVVAVGYGTENGKDYWIVR
NSWGNRWGESGYIKMARNIEAPTGKCGIAMEASYPIKKGQNPPNPGPSPPSPIKPPTTCDKYFSCPESNTCCCLYKYGKY
CFGWGCCPLEAATCCDDNSSCCPHEYPVCDVNRGTCLMSKNSPFSVKALKRTPAIPFWAKSRKHIA*
>AtRD21C
MATSIKSITLALLIFSVLLISLSLGSVTATETTRNEAEARRMYERWLVENRKNYNGLGEKERRFEIFKDNLKFVEEHSSIPNR
TYEVGLTRFADLTNDEFRAIYLRSKMERTRVPVKGEKYLYKVGDSLPDAIDWRAKGAVNPVKDQGSCGSCWAFSAIGAV
EGINQIKTGELISLSEQELVDCDTSYNDGCGGGLMDYAFKFIIENGGIDTEEDYPYIATDVNVCNSDKKNTRVVTIDGYED
VPQNDEKSLKKALANQPISVAIEAGGRAFQLYTSGVFTGTCGTSLDHGVVAVGYGSEGGQDYWIVRNSWGSNWGESGY
FKLERNIKESSGKCGVAMMASYPTKSSGSNPPKPPAPSPVVCDKSNTCPAKSTCCCLYEYNGKCYSWGCCPYESATCCDD
GSSCCPQSYPVCDLKANTCRMKGNSPLSIKALTRGPAIATTKSTNMLVGSA*
>AtRDL1
MAPSTKVLSLLLLYVVVSLASGDESIINDHLQLPSDGKWRTDEEVRSIYLQWSAEHGKTNNNNNGIINDQDKRFNIFKDN
LRFIDLHNENNKNATYKLGLTKFTDLTNDEYRKLYLGARTEPARRIAKAKNVNQKYSAAVNGKEVPETVDWRQKGAVN
PIKDQGTCGSCWAFSTTAAVEGINKIVTGELISLSEQELVDCDKSYNQGCNGGLMDYAFQFIMKNGGLNTEKDYPYRGFG
GKCNSFLKNSRVVSIDGYEDVPTKDETALKKAISYQPVSVAIEAGGRIFQHYQSGIFTGSCGTNLDHAVVAVGYGSENGVD
YWIVRNSWGPRWGEEGYIRMERNLAASKSGKCGIAVEASYPVKYSPNPVRGNTISSV*
>AtRDL2

MAATPIRVIVSALVILSVLLLSSSLGVATETEIERNETEVRLMYEQWLVENRKNYNGLGEKERRFKIFKDNLKFVDEHNSV
PDRTFEVGLTRFADLTNEEFRAIYLRKKMERTKDSVKTERYLYKEGDVLPDEVDWRANGAVVSVKDQGNCGSCWAFSAV
GAVEGINQITTGELISLSEQELVDCDRGFVNAGCDGGIMNYAFEFIMKNGGIETDQDYPYNANDLGLCNADKNNNTRVVT
IDGYEDVPRDDEKSLKKAVAHQPVSVAIEASSQAFQLYKSGVMTGTCGISLDHGVVVVGYGSTSGEDYWIIRNSWGLNW
GDSGYVKLQRNIDDPFGKCGIAMMPSYPTKSSFPSSFDLLSEI*
>AtRDL3
MAISFRTLALLTLSVLLISISLGVVTATESQRNEGEVLTMYEQWLVENGKNYNGLGEKERRFKIFKDNLKRIEEHNSDPNR
SYERGLNKFSDLTADEFQASYLGGKMEKKSLSDVAERYQYKEGDVLPDEVDWRERGAVVPRVKRQGECGSCWAFAATG
AVEGINQITTGELVSLSEQELIDCDRGNDNFGCAGGGAVWAFEFIKENGGIVSDEVYGYTGEDTAACKAIEMKTTRVVTIN
GHEVVPVNDEMSLKKAVAYQPISVMISAANMSDYKSGVYKGACSNLWGDHNVLIVGYGTSSDEGDYWLIRNSWGPEW
GEGGYLRLQRNFHEPTGKCAVAVAPVYPIKSNSSSHLLSPSVFKLVVLFVFQLISLALL*
>AtRDL4
MGSAKSAMLILLVAMVIASCATAIDMSVVSYDDNNRLHSVFDAEASLIFESWMVKHGKVYGSVAEKERRLTIFEDNLRFI
NNRNAENLSYRLGLTGFADLSLHEYKEVCHGADPRPPRNHVFMTSSDRYKTSADDVLPKSVDWRNEGAVTEVKDQGHC
RSCWAFSTVGAVEGLNKIVTGELVTLSEQDLINCNKENNGCGGGKLETAYEFIMKNGGLGTDNDYPYKAVNGVCDGRLK
ENNKNVMIDGYENLPANDESALMKAVAHQPVTAVIDSSSREFQLYESGVFDGSCGTNLNHGVVVVGYGTENGRDYWLV
KNSRGITWGEAGYMKMARNIANPRGLCGIAMRASYPLKNSFSTDKSSIA*
>AtRDL5
MGYAKSAMLIFLLALVIASCATAMDMSVVSSNDNHHVTAGPGRRQGIFDAEATLMFESWMVKHGKVYDSVAEKERRLTI
FEDNLRFITNRNAENLSYRLGLNRFADLSLHEYGEICHGADPRPPRNHVFMTSSNRYKTSDGDVLPKSVDWRNEGAVTE
VKDQGLCRSCWAFSTVGAVEGLNKIVTGELVTLSEQDLINCNKENNGCGGGKVETAYEFIMNNGGLGTDNDYPYKALN
GVCEGRLKEDNKNVMIDGYENLPANDEAALMKAVAHQPVTAVVDSSSREFQLYESGVFDGTCGTNLNHGVVVVGYGTE
NGRDYWIVKNSRGDTWGEAGYMKMARNIANPRGLCGIAMRASYPLKNSFSTDKVSVA*
>AtRDL6
MGFVRPVCMTILFLLIVFVLSAPSSAMDLPATSGGHNRSNEEVEFIFQMWMSKHGKTYTNALGEKERRFQNFKDNLRFID
QHNAKNLSYQLGLTRFADLTVQEYRDLFPGSPKPKQRNLKTSRRYVPLAGDQLPESVDWRQEGAVSEIKDQGTCNSCWA
FSTVAAVEGLNKIVTGELISLSEQELVDCNLVNNGCYGSGLMDTAFQFLINNNGLDSEKDYPYQGTQGSCNRKQSTSNKV
ITIDSYEDVPANDEISLQKAVAHQPVSVGVDKKSQEFMLYRSCIYNGPCGTNLDHALVIVGYGSENGQDYWIVRNSWGTT
WGDAGYIKIARNFEDPKGLCGIAMLASYPIKNSASNA*
>AtXCP1
MAFSAPSLSKFSLLVAISASALLCCAFARDFSIVGYTPEHLTNTDKLLELFESWMSEHSKAYKSVEEKVHRFEVFRENLMH
IDQRNNEINSYWLGLNEFADLTHEEFKGRYLGLAKPQFSRKRQPSANFRYRDITDLPKSVDWRKKGAVAPVKDQGQCGS
CWAFSTVAAVEGINQITTGNLSSLSEQELIDCDTTFNSGCNGGLMDYAFQYIISTGGLHKEDDYPYLMEEGICQEQKEDVE
RVTISGYEDVPENDDESLVKALAHQPVSVAIEASGRDFQFYKGGVFNGKCGTDLDHGVAAVGYGSSKGSDYVIVKNSWG
PRWGEKGFIRMKRNTGKPEGLCGINKMASYPTKTK*
>AtXCP2
MALSSPSRILCFALALSAASLSLSFASSHDYSIVGYSPEDLESHDKLIELFENWISNFEKAYETVEEKFLRFEVFKDNLKHID
ETNKKGKSYWLGLNEFADLSHEEFKKMYLGLKTDIVRRDEERSYAEFAYRDVEAVPKSVDWRKKGAVAEVKNQGSCGS
CWAFSTVAAVEGINKIVTGNLTTLSEQELIDCDTTYNNGCNGGLMDYAFEYIVKNGGLRKEEDYPYSMEEGTCEMQKDE
SETVTINGHQDVPTNDEKSLLKALAHQPLSVAIDASGREFQFYSGGVFDGRCGVDLDHGVAAVGYGSSKGSDYIIVKNS
WGPKWGEKGYIRLKRNTGKPEGLCGINKMASFPTKTK*
>AtXBCP3
MSMSSSSFISLTFFFLLLVSSSSSSDDISELFDDWCQKHGKTYGSEEERQQRIQIFKDNHDFVTQHNLITNATYSLSLNAFAD
LTHHEFKASRLGLSVSAPSVIMASKGQSLGGSVKVPDSVDWRKKGAVTNVKDQGSCGACWSFSATGAMEGINQIVTGD
LISLSEQELIDCDKSYNAGCNGGLMDYAFEFVIKNHGIDTEKDYPYQERDGTCKKDKLKQKVVTIDSYAGVKSNDEKAL
MEAVAAQPVSVGICGSERAFQLYSSGIFSGPCSTSLDHAVLIVGYGSQNGVDYWIVKNSWGKSWGMDGFMHMQRNTEN
SDGVCGINMLASYPIKTHPNPPPPSPPGPTKCNLFTYCSSGETCCCARELFGLCFSWKCCEIESAVCCKDGRHCCPHDYPV
CDTTRSLCLKKTGNFTAIKPFWKKNSSKQLGRFEEWVM*
>AtTHI1

MLNVLRNSNLTLAVLICFVLIASKLCSVDSSVYDPHKTLKQRFEKWLKTHSKLYGGRDEWMLRFGIYQSNVQLIDYINSL
HLPFKLTDNRFADMTNSEFKAHFLGLNTSSLRLHKKQRPVCDPAGNVPDAVDWRTQGAVTPIRNQGKCGGCWAFSAVAA
IEGINKIKTGNLVSLSEQQLIDCDVGTYNKGCSGGLMETAFEFIKTNGGLATETDYPYTGIEGTCDQEKSKNKVVTIQGYQ
KVAQNEASLQIAAAQQPVSVGIDAGGFIFQLYSSGVFTNYCGTNLNHGVTVVGYGVEGDQKYWIVKNSWGTGWGEEG
YIRMERGVSEDTGKCGIAMMASYPLQ*
>AtSAG12
MALKHMQIFLFVAIFSSFCFSITLSRPLDNELIMQKRHIEWMTKHGRVYADVKEENNRYVVFKNNVERIEHLNSIPAGRTF
KLAVNQFADLTNDEFRSMYTGFKGVSALSSQSQTKMSPFRYQNVSSGALPVSVDWRKKGAVTPIKNQGSCGCCWAFSAV
AAIEGATQIKKGKLISLSEQQLVDCDTNDFGCEGGLMDTAFEHIKATGGLTTESNYPYKGEDATCNSKKTNPKATSITGYE
DVPVNDEQALMKAVAHQPVSVGIEGGGFDFQFYSSGVFTGECTTYLDHAVTAIGYGESTNGSKYWIIKNSWGTKWGESG
YMRIQKDVKDKQGLCGLAMKASYPTI*
>AtPAP1
MASIMVLVTVLIILFTGFRISQATSRTVIFREQSMVDKHEQWMARFSREYRDELEKNMRRDVFKKNLKFIENFNKKGNKS
YKLGVNEFADWTNEEFLAIHTGLKGLTEVSPSKVVAKTISSQTWNVSDMVVESKDWRAEGAVTPVKYQGQCGCCWAFS
AVAAVEGVAKIAGGNLVSLSEQQLLDCDREYDRGCDGGIMSDAFNYVVQNRGIASENDYSYQGSDGGCRSNARPAARIS
GFQTVPSNNERALLEAVSRQPVSVSMDATGDGFMHYSGGVYDGPCGTSSNHAVTFVGYGTSQDGTKYWLAKNSWGET
WGEKGYIRIRRDVAWPQGMCGVAQYAFYPVA*
>AtPAP2
MCDSFINNKMTSILFMLVSLTILSMNLKVSQATSRVTFHEPIVAEHHQQWMTRFSRVYSDELEKQMRFDVFKKNLKFIEKF
NKKGDRTYKLGVNEFADWTREEFIATHTGLKGVNGIPSSEFVDEMIPSWNWNVSDVAGRETKDWRYEGAVTPVKYQGQ
CGCCWAFSSVAAVEGLTKIVGNNLVSLSEQQLLDCDRERDNGCNGGIMSDAFSYIIKNRGIASEASYPYQAAEGTCRYNG
KPSAWIRGFQTVPSNNERALLEAVSKQPVSVSIDADGPGFMHYSGGVYDEPYCGTNVNHAVTFVGYGTSPEGIKYWLAK
NSWGETWGENGYIRIRRDVAWPQGMCGVAQYAFYPVA*
>AtPAP3
MDFVEFVCVVLTIFFMDLKISEATSRVALYKPSSIVDYHQQWMIQFSRVYDDEFEKQLRLQVLTENLKFIESFNNMGNQSY
KLGVNEFTDWTKEEFLATYTGLRGVNVTSPFEVVNETKPAWNWTVSDVLGTNKDWRNEGAVTPVKSQGECGGCWAFS
AIAAVEGLTKIARGNLISLSEQQLLDCTREQNNGCKGGTFVNAFNYIIKHRGISSENEYPYQVKEGPCRSNARPAILIRGFE
NVPSNNERALLEAVSRQPVAVAIDASEAGFVHYSGGVYNARNCGTSVNHAVTLVGYGTSPEGMKYWLAKNSWGKTWG
ENGYIRIRRDVEWPQGMCGVAQYASYPVA*
>AtPAP4
MASTIIFILTIFLSYRTSLATSRGSLFEASAIEKHEQWMARFNRVYSDETEKRNRFNIFKKNLEFVQNFNMNNKITYKVDIN
EFSDLTDEEFRATHTGLVVPEAITRISTLSSGKNTVPFRYGNVSDNGESMDWRQEGAVTPVKYQGRCGGCWAFSAVAAVE
GITKITKGELVSLSEQQLLDCDRDYNQGCRGGIMSKAFEYIIKNQGITTEDNYPYQESQQTCSSSTTLSSSFRAATISGYETV
PMNNEEALLQAVSQQPVSVGIEGTGAAFRHYSGGVFNGECGTDLHHAVTIVGYGMSEEGTKYWVVKNSWGETWGENG
YMRIKRDVDAPQGMCGLAILAFYPLA*
>AtPAP5
MTSIVFFLLAILLSSRTSGVTSRGGLFEASAVEKHEQWMSRFNRVYSDDSEKTSRFEIFTNNLKFVESINMNTNKTYTLDV
NEFSDLTDEEFKARYTGLVVPEGMTRISTTDSHETVSFRYENVGETGESMDWIQEGAVTSVKHQQQCGCCWAFSAVAAVE
GMTKIANGELVSLSEQQLLDCSTENNGCGGGIMWKAFDYIKENQGITTEDNYPYQGAQQTCESNHLAAATISGYETVPQ
NDEEALLKAVSQQPVSVAIEGSGYEFIHYSGGIFNGECGTQLTHAVTIVGYGVSEEGIKYWLLKNSWGESWGENGYMRIM
RDVDSPQGMCGLASLAYYPVA*
>AtRD19A
MDRLKLYFSVFVLSFFIVSVSSSDVNDGDDLVIRQVVGGAEPQVLTSEDHFSLFKRKFGKVYASNEEHDYRFSVFKANLR
RARRHQKLDPSATHGVTQFSDLTRSEFRKKHLGVRSGFKLPKDANKAPILPTENLPEDFDWRDHGAVTPVKNQGSCGSC
WSFSATGALEGANFLATGKLVSLSEQQLVDCDHECDPEEADSCDSGCNGGLMNSAFEYTLKTGGLMKEEDYPYTGKDG
KTCKLDKSKIVASVSNFSVISIDEEQIAANLVKNGPLAVAINAGYMQTYIGGVSCPYICTRRLNHGVLLVGYGAAGYAPAR
FKEKPYWIIKNSWGETWGENGFYKICKGRNICGVDSMVSTVAATVSTTAH*
>AtRD19B
MDYHLRVLFSVSLIFVFVSVSVCGDEDVLIRQVVDETEPKVLSSEDHFTLFKKKFGKVYGSIEEHYYRFSVFKANLLRAM

RHQKMDPSARHGVTQFSDLTRSEFRRKHLGVKGGFKLPKDANQAPILPTQNLPEEFDWRDRGAVTPVKNQGSCGSCWS
FSTTGALEGAHFLATGKLVSLSEQQLVDCDHECDPEEEGSCDSGCNGGLMNSAFEYTLKTGGLMREKDYPYTGTDGGSC
KLDRSKIVASVSNFSVVSINEDQIAANLIKNGPLAVAINAAYMQTYIGGVSCPYICSRRLNHGVLLVGYGSAGFSQARLKE
KPYWIIKNSWGESWGENGFYKICKGRNICGVDSLVSTVAATTS*
>AtRD19C
MDRVVFFFLIAATLLAGSLGSTVISGEVTDGFVNPIRQVVPEENDEQLLNAEHHFTLFKSKYEKTYATQVEHDHRFRVFKA
NLRRARRNQLLDPSAVHGVTQFSDLTPKEFRRKFLGLKRRGFRLPTDTQTAPILPTSDLPTEFDWREQGAVTPVKNQGMC
GSCWSFSAIGALEGAHFLATKELVSLSEQQLVDCDHECDPAQANSCDSGCSGGLMNNAFEYALKAGGLMKEEDYPYTG
RDHTACKFDKSKIVASVSNFSVVSSDEDQIAANLVQHGPLAIAINAMWMQTYIGGVSCPYVCSKSQDHGVLLVGFGSSG
YAPIRLKEKPYWIIKNSWGAMWGEHGYYKICRGPHNMCGMDTMVSTVAAVHTSPK*
>AtRD19D
MVAKALAQLITCIILFCHVVASVEDLTIRQVTADNRRIRPNLLGTHTESKFRLFMSDYGKNYSTREEYIHRLGIFAKNVLKA
AEHQMMDPSAVHGVTQFSDLTEEEFKRMYTGVADVGGSRGGTVGAEAPMVEVDGLPEDFDWREKGGVTEVKNQGAC
GSCWAFSTTGAAEGAHFVSTGKLLSLSEQQLVDCDQACDPKDKKACDNGCGGGLMTNAYEYLMEAGGLEEERSYPYT
GKRGHCKFDPEKVAVRVLNFTTIPLDENQIAANLVRHGPLAVGLNAVFMQTYIGGVSCPLICSKRNVNHGVLLVGYGSKG
FSILRLSNKPYWIIKNSWGKKWGENGYYKLCRGHDICGINSMVSAVATQVSS*
>AtAALP
MSAKTILSSVVLVVLVAASAAANIGFDESNPIRMVSDGLREVEESVSQILGQSRHVLSFARFTHRYGKKYQNVEEMKLRF
SIFKENLDLIRSTNKKGLSYKLGVNQFADLTWQEFQRTKLGAAQNCSATLKGSHKVTEAALPETKDWREDGIVSPVKDQ
GGCGSCWTFSTTGALEAAYHQAFGKGISLSEQQLVDCAGAFNNYGCNGGLPSQAFEYIKSNGGLDTEKAYPYTGKDETC
KFSAENVGVQVLNSVNITLGAEDELKHAVGLVRPVSIAFEVIHSFRLYKSGVYTDSHCGSTPMDVNHAVLAVGYGVEDG
VPYWLIKNSWGADWGDKGYFKMEMGKNMCGIATCASYPVVA*
>AtALP2
MSVKLNLSSSILLILFAAAASKEIGFDESNPIKMVSDNLHELEDTVVQILGQSRHVLSFSRFTHRYGKKYQSVEEMKLRFS
VFKENLDLIRSTNKKGLSYKLSLNQFADLTWQEFQRYKLGAAQNCSATLKGSHKITEATVPDTKDWREDGIVSPVKEQG
HCGSCWTFSTTGALEAAYHQAFGKGISLSEQQLVDCAGTFNNFGCHGGLPSQAFEYIKYNGGLDTEEAYPYTGKDGGCK
FSAKNIGVQVRDSVNITLGAEDELKHAVGLVRPVSVAFEVVHEFRFYKKGVFTSNTCGNTPMDVNHAVLAVGYGVEDD
VPYWLIKNSWGGEWGDNGYFKMEMGKNMCGVATCSSYPVVA*
>AtCTB1
MADSCCIRLHLLASVFLLLFSSFNLQGIAAENLSKQKLTSLILQNEIVKEVNENPNAGWKAAFNDRFANATVAEFKRLLGV
IQTPKTAYLGVPIVRHDLSLKLPKEFDARTAWSHCTSIRRILVGYILNNVLLWSTITLWFWFLLGHCGSCWAFGAVESLSDR
FCIKYNLNVSLSANDVIACCGLLCGFGCNGGFPMGAWLYFKYHGVVTQECDPYFDNTGCSHPGCEPTYPTPKCERKCVS
RNQLWGESKHYGVGAYRINPDPQDIMAEVYKNGPVEVAFTVYEDFAHYKSGVYKYITGTKIGGHAVKLIGWGTSDDGE
DYWLLANQWNRSWGDDGYFKIRRGTNECGIEQSVVAGLPSEKNVFKGITTSDDLLVSSV*
>AtCTB2
MADNCIRLLHSASVFFCLGLLISSFNLLQGIAAENLSKQKLTSWILQNEIVKEVNENPNAGWKASFNDRFANATVAEFKRL
LGVKPTPKTEFLGVPIVSHDISLKLPKEFDARTAWSQCTSIGRILDQGHCGSCWAFGAVESLSDRFCIKYNMNVSLSVNDL
LACCGFLCGQGCNGGYPIAAWRYFKHHGVVTEECDPYFDNTGCSHPGCEPAYPTPKCARKCVSGNQLWRESKHYGVSA
YKVRSHPDDIMAEVYKNGPVEVAFTVYEDFAHYKSGVYKHITGTNIGGHAVKLIGWGTSDDGEDYWLLANQWNRSWG
DDGYFKIRRGTNECGIEHGVVAGLPSDRNVVKGITTSDDLLVSSF*
>AtCTB3
MAVYNTKLCLASVFLLLGLLLAFDLKGIEAESLTKQKLDSKILQDEIVKKVNENPNAGWKAAINDRFSNATVAEFKRLLG
VKPTPKKHFLGVPIVSHDPSLKLPKAFDARTAWPQCTSIGNILDQGHCGSCWAFGAVESLSDRFCIQFGMNISLSVNDLLA
CCGFRCGDGCDGGYPIAAWQYFSYSGVVTEECDPYFDNTGCSHPGCEPAYPTPKCSRKCVSDNKLWSESKHYSVSTYTV
KSNPQDIMAEVYKNGPVEVSFTVYEDFAHYKSGVYKHITGSNIGGHAVKLIGWGTSSEGEDYWLMANQWNRGWGDDG
YFMIRRGTNECGIEDEPVAGLPSSKNVFRVDTGSNDLPVASV*
>VvXCP1
MALLSSFSKVSILALSLSLLAFSSLARDFSIVGYSPEDLTCIDKLIARFESWVSKHGKVYKSMEEKLHRFEVFRENLNHIDE
RNKEVSSYWLGLNEFADLSHEEFKSKYLGLRAEFPRSRDYSGEFRYRDVADLPESVDWRKKGAVTHVKNQGACGSCWA

FSTVAAVEGINQIVTGNLTTLSEQELIDCDTTFNSGCNGGLMDYAFAFIASNGGLHKEDDYPYLMEEGTCEEQKEDVDIVT
ISGYEDVPEKDEESLLKALAHQPLSVAIEASGRDFQFYSGGVFNGPCGTELDHGVAAVGYGSSKGLDYIIVKNSWGPKWG
EKGYIRMKRNTGKTEGLCGINKMASYPTKDN*
>VvXCP2
MALSPFSNFFLLFISMAVFAYSAFARDFSIVGYSPDDLTSMDKLTDLFESWMSKHGKSYRSFEEKLHRFEVFQDNLKHIDE
TNKKVSSYWLGLNEFADLSHEEFKRKYLGLKIELPKRRDSPEEFSYKDVADLPKSVDWRKKGAVAHVKNQGACGSCWA
FSTVAAVEGINQIVTGNLTALSEQELIDCDKPFNNGCNGGLMDYAFAFIISNGGLRKEEDYPYVMEEGTCGEKKEELEVVT
ISGYHDVPEDNEQSFLKALANQPLSVAIEASSRGFQFYSGGIFNGHCGTELDHGVAAVGYGTSKGVDYITVKNSWGSKW
GEKGYIRMKRNVGKPEGICGIYKMASYPTKNK*
>VvRD21-1
MGLCRSSSSMAVFLFLLLGLASALDMSIIGYDETHGDKSSWRTDEDVMAVYEAWLAKHGKSYNALGEKERRFQIFKDNL
RFIDEHNAENRTYKVGLNRFADLTNEEYRSMYLGTRTAAKRRSSNKISDRYAFRVGDSLPESVDWRKKGAVVEVKDQGS
CGSCWAFSTIAAVEGINKIVTGGLISLSEQELVDCDTSYNEGCNGGLMDYAFEFIINNGGIDSEEDYPYKASDGRCDQYRK
NAKVVTIDGYEDVPENDEKSLEKAVANQPVSVAIEAGGREFQLYQSGIFTGRCGTALDHGVTAVGYGTENGVDYWIVKN
SWGASWGEEGYIRMERDLATSATGKCGIAMEASYPIKKGQNPPNPGPSPPSPIKPPTVCDNYYACPESSTCCCIFEYAKYC
FQWGCCPLEAATCCEDHDSCCPQEYPVCNVRAGTCMMSKDNPLGVKALKRTAAKPHWAYGGDGKRSSA*
>VvRD21-2
MAVSQSPIMASFLFSFFFLLAALFSASASAADMSIISYGDRLEKRTDAEVMAVYEAWLVKHGKSYNALGERERRFEIFKD
NLRFIEEHNAVNRTYKVGLNRFADLTNEEYRSRYLGRRDETRRGLRASRVSDRYSFRAGEDLPESVDWREKGAVVPVKD
QGNCGSCWAFSTIAAVEGINQIATGDLISLSEQELVDCDKSYNQGCNGGLMDYAFEFIINNGGIDSEEDYPYRAADTTCDP
NRKNARVVSIDGYEDVPQNDERSLKKAVANQPVSVAIEAGGRAFQLYQSGVFTGQCGTQLDHGVVAVGYGTENSVDYW
IVRNSWGPNWGESGYIKLERNLAGTETGKCGIAIEPSYPIKNGQNPPNPGPSPPSPSKPSVVCDEYYTCPEESTCCCIYEYA
GFCFEWGCCPLEGATCCDDHYSCCPHEYPVCDVDAGTCQMSKGNPLSVKAWRRTPARPVFAPRPVIEVI*
>VvRD21-3
MISSTSSRLPAMALSISTLLFLFFVASSAADLSSSWRSEEEVMGMYQWWMAKHGKAYNGLGEKEKRFEIFKDNLKFIDEH
NAQNRTYKVGLNRFADLTNEEYRAIYLGTRSDPKRRFAKLKNASPRYAVMPGEVLPESVDWRETGAVNPVKDQRSCGSC
WAFSTVAAVEGINQIVTGELISLSEQELVDCDTEYDMGCNGGLMDYAFDFIIKNGGLDTEKDYPYTGFDGECNLSGSSKV
VSIDGYEDVPPFDEKALQKAVAHQPVSVAVEAGGRALQLYVSGIFTGECGTALDHGIVAVGYGTENGTDYWIVRNSWGSS
WGENGYIRMERNMADAFSGKCGIAMEASYPIKNGENPSKTYLSFGTAGEKISSA*
>VvCEP1
MPIRNLQLLHLLSLVLFLHSHLLSSIWLFFHSSIYTWFRVSQLPSKYQKMKRFFVVALSLVLVVGIVESFDFHQKELETEES
LWNLYERWRSHHTVSRSLDEKHKRFNVFKENVNFVHEFNKKDEPYKLKLNKFADMTNHEFRSTYAGSKVNHHRMFRG
SQHAAGSFMYEKVKSVPPSVDWRKKGAVTPIKDQGQCGSCWAFSTVVAVEGINHIKTNKLVSLSEQELVDCDTSENQGC
NGGLMGYAFEFIKEKGGITTEQSYPYTAEDGTCDVSKVNSPVVSIDGHETVPPNNEDALLKAAANQPISVAIDAGGSAFQF
YSEGVFAGRCGTDLDHGVAIVGYGTTLDGTKYWIVKNSWGTDWGENGYIRMKRGISAKEGLCGIAVEASYPIKNSSTNP
VGAPSSLKDEL*
>VvSAG12-3
MASVNQYQYICLALLFVLAAWASQATARNLHEASMYERHEDWMAQYGRVYKDADEKSKRYKIFKDNVARIESFNKAM
DKSYKLSINEFADLTNEEFGTSRNRFKAHICSTEATSFKYENVTAVPSTIDWRKKGAVTPIKDQGQCGSCWAFSAVAAMEG
ITQLSTGKLISLSEQELVDCDTSGEDQGCNGGLMDDAFKFIEQNHGLATEANYPYAGTDGTCNRKKAAHPAAKINGYED
VPANNEKALQKAVAHQPIAVAIDAGGFEFQFYSSGVFTGQCGTELDHGVAAVGYGTSDDGMKYWLVKNSWGTGWGEV
GYIRMQRDVTAKEGLCGIAMQASYPTA*
>VvSAG12-2
MASVNQYQYICLALLFVLAAWASQATARSLHEASMYERHEDWMVQYGREYKDADEKSKRYKIFKDNVARIESFNKAM
DKSYKLSINEFADLTNEEFRASRNRFKAHICSTEATSFKYENVTAVPSTVDWRKKGAVTPIKDQGQCGSCWAFSAVAAME
GITQLSTGKLISLSEQELVDCDTSGEDQGCSGGLMDDAFKFIEQNHGLTTEANYPYAGTDGTCNRKKAAHPAAKINGYED
VPANNEKALQKAVAHQPIAVAIDASGSEFQFYSSGVFTGQCGTELDHGVAAVGYGTSDDGMKYWLVKNSWSTGWGEEG
YIRMQRDVTAKEGLCGIAMQASYPTA*
>VvSAG12-1

MASVNQYQYICLALLFVLAAWASQATARNLHEASMYERHEDWMVQYGREYKDADEKSKRYKIFKDNVARIESFNKAM
DKSYKLSINEFADLTNEEFRASRNRFKAHICSTEATSFKYENVTAVPSTVDWRKKGAVTPIKDQGQCGSCWAFSAVAAME
GITQLSTGKLISLSEQELVDCDTSGEDQGCSGGLMDDAFKFIEQNHGLTTEANYPYAGTDGTCNRKKAAHPAAKINGYED
VPANNEKALQKAVAHQPIAVAIDAGGSEFQFYSSGVFTGQCGTELDHGVSAVGYGTSDDGMKYWLVKNSWGTGWGEE
GYIRMQRDVTAKEGLCGIAMQASYPTA*

>VvCEP2

MKMEKVILVALSLVLVFGLAESFDFDEKDLASEESLWDLYERWRSYHTVSRDLEEKNKRFNVFKENTKHVHKVNQMDK
PYKLKLNKFADMTNHEFRSSYGGSKVKHYRMLRGDRRGTGGFMHEKTTYLPPSVDWRKKGAVTGIKDQGKCGSCWAF
STVVGVEGINQIKTKELLSLSEQQLIDCDRSDDHGCNGGLMESAFEFIKKNGGITTENNYPYKAKDERCDMLKMNAPVV
TIDGHESVPVNDERALMKAVAHQPVSVAIDAGGSDLQFYSEGVFDGECGTELDHGVAIVGYGTTLDGTKYWIVKNSWG
AEWGEKGYIRMARGIQAAEGQCGIAMEASYPVKSSNNTRRGSIKDEL*

>VvSAG12-4

MFLLLRTSIMASTNQYQYVSMALLFILAAWASQATSRSLHEASMYERHEDWMARYGRMYKDANEKEKRFKIFKDNVAR
IESFNKAMDKTYKLSINEFADLTNEEFRSLRNRFKAHICSEATTFKYENVTAVPSTIDWRKKGAVTPIKDQQQCGCCWAFS
AVAATEGITQITTGKLISLSEQELVDCDTGGENQGCSGGLMDDAFRFIKIHGLASEATYPYEGDDGTCNSKKEAHPAAKIK
GYEDVPANNEKALQKAVAHQPVAVAIDAGGFEFQFYTSGVFTGQCGTELDHGVAAVGYGIGDDGMMYWLVKNSWGTG
WGEEGYIRMQRDVTAKEGLCGIAMQASYPTA*

>VvSAG12-5

MALESKIICITLLIMGVWASQALSRTLHEVSMSERHEDWMGLYGRTYKDIAEKERRFKIFKENVEYIESVNSAGNRRYKLS
INEFADQTNEEFKASRNGYNMSSRPRSSEITSFRYENVAAVPSSMDWRKKGAVTPIKDQGQCGCCWAFSAVAAMEGVTQ
LKTGELISLSEQELVDCDTSGEDQGCGGGLMDSAFEFIIGNGGLTTEANYPYKGVDATCNKKKAASSAAKIKNYEDVPAN
SEAALLKAVAQHPVSVAIDAGGSDFQFYSSGVFTGQCGTELDHGVTAVGYGKTDDGTKYWLVKNSWGTGWGEDGYIW
MERDIGADEGLCGIAMEASYPTA*

>VvXBCP1

MGSWLWAVSILILAVHSSVSEASSTADLFEAWCEQYGKTYSSEEEKASRLKVFEENHAFVTQHNSMANASYTLALNAFA
DLTHHEFKASRLGFSPGRAQSIRSVGTPVQELHVPPAVDWRKSGAVTGVKDQGNCGGCWSFSTTGAIEGINKIVTGSLVSL
SEQELVDCDRSYNSGCEGGLMDYAYQFVIKNQGIDSEADYPYVGMDKPCNKEKLKKHIVTIDGYTDIPPNDEKQLLQVV
AKQPVSVGICGSEKTFQLYSKGVYTGPCSSTLDHAVLIVGYGTEDGVDFWIVKNSWGEHWGMRGYIHMLRNNGTAEGI
CGINMLASYPAKTSPNPPPPPTPGPTKCDFFSSCSEGETCCCSWRFIGVCLSWNCCTAKSAVCCDNNNYCCPASHPICDTK
RNRCLKPAGNGTGVEVLKRRGSSVKFGGWSSINDAWNL*

>VvTHI1

MKTSMFCRNVYFALLIMWTVGVSWSAFSEEHEPMESEMSDMEKRYERWLVQHGRRYKNRDEWQRHFGIYQSNVRFIN
YINAQNFSFTLTDNQFADMTNEEYKALYMGLGTSETSRKNQSSFKRERSKVLPISVDWRKMGAVTPVRNQGECGSCWAF
STVAAVEGINKIRTGKLVSLSEQELLDCDIDSGNEGCNGGYMVNAFKFIKQNGGITTARNYPYIGEQGICNKDKAANHVV
KISGYETVPPNNEKILQAAVAKQPVSVAIDAGGYEFQLYSKGIFNGFCGKQLNHAVTVIGYGEDNGKKYWLVKNSWGTG
WGEAGYARMIRDSRDDEGICGIAMEASYPIKAVSLVGEAGSSS*

>VvXBCP2

MGSQKIQLALVLFIWASLACLSSSLPTEFYITGEEFASEERVRELFHLWKERHKRVYKHAEETAKRFEIFKENLKYVIERNS
KGHRHTLGMNKFADMSNEEFKEKYLSKIKKPINKKNNYLRRSMQQKKGTASCEAPSSLDWRKKGVVTGIKDQGDCGSC
WAFSSTGAMEGINAIVTGDLISLSEQELVDCDTTNYGCEGGYMDYAFEWVISNGGIDSESDYPYTGTDGTCNTTKEDTKV
VSIDGYKDVDESDSALLCAAVNQPISVGMDGSALDFQLYTSGIYAGDCSDDPDDIDHAVLIVGYGSEDSEDYWICKNSWG
TSWGMEGYFYIKRNTDLPYGECAINAMASYPTKESSSPSPYPSPAVPPPPPPPPSPPPPPPPSPPPPSPGPSPSECGDFSYCPS
DETCCCIYEFYDFCLIYGCCEYENAVCCTGTEYCCPSDYPICDVEEGLCLKNQGDYLGVAAKKRKMAKHKFPWTKIEET
QKTYQPLEWKRNRFAAMR*

>VvALP1

MARLSVVAAVLILLCAVASGEADHHFRSSFDEENPIRLVSDSIRDLESSVLRLIGDTRHAHSFASFAHRYGKSYKTVDEIKL
RFEIFSENLKLIRSTNRKGLPYTLAVNQFADWTWEEFRRHRLGAAQNCSATLKGNHKLTDVILPETKDWREDGIVSPIKDQ
GHCGSCWTFSTTGALEAAYAQAFGKGISLSEQQLVDCAGAFNNFGCHGGLPSQAFEYIKYNGGLDTEEAYPYTGLDGTC
KFSSENIGVQVLDSVNITLGAEDELKHAVAFVRPVSVAFEVVHDFRFYKKGVYTSGTCGSTPMDVNHAVLAVGYGVEDG

VAYWLIKNSWGENWGDNGYFKMELGKNMCGVATCSSYPVVA*
>VvRD19-1
MDRLSCFCLFLFLSSLLFSAAVSVSVSDVSFDESEDLLIHQVVSDGDDLLNAEYQFAEFKTKFGKTYATAEEHDHRFNVFK
ANLRRAKRHQLLDPSAEHGVTQFSDLTPREFRQNYLGLKRLQLPADAQKAPILPTKDLPTDFDWRDHGAVTAVKDQGYC
GSCWSFSTIGALEGAHFLATGNLVSLSTQQLLDCDTECDPEEYDACDDGCNGGLMNNAFEYILKAGGVAQEEDYPYTGT
DRGLCRFNKTKIAASVANFSVVSLDEDQIAANLVKNGPLAVGINAVFMQTYKSGVSCPYICSSTLDHGVLLVGYGSAGYS
PIRFKEKPYWIIKNSWGESWGEQGYYKICRGHNICGVDSMVSTVAAIHTTAL*
>VvRD19-3
MARSFSLLFFLLFSLFFVALTSSELHSGGSDDDIIIRQVVPELGDVEGSEEENLLTADHHHFSIFKRRFGKSYASQEEHDYRF
KVFKANLRRARRHQQLDPSATHGVTQFSDLTPAEFRGTYLGLRPLKLPHDAQKAPILPTNDLPEDFDWRDHGAVTAVKN
QGSCGSCWSFSTTGALEGANFLATGNLVSLSEQQLVECDHECDPEEMGSCDSGCNGGLMNTAFEYTLKAGGLMKEEDY
PYTGTDRGSCKFDKTKIAASVSNFSVISLDEDQIAANLVKNGPLAVAINAVFMQTYVGGVSCPYICSKRLDHGVLLVGYGS
AGYAPIRMKDKPYWIIKNSWGENWGENGFYKICRGRNVCGVDSMVSTVAAVHTTSN*
>VvRD19-2
MDFLSNFVLLLLVPSLLVLKAVSEASFDESDNLMIRQVESHVDDFLNAERHFEKFKARFQKTYATPEEHDYRFNVFKANL
RRAKRHQLLDPSAVHGVTQFSDLTPAEFRRDYLGLNPLRFPADAQQAPILPTDNLPTDFDWRENGAVTPVKNQGNCGSC
WSFSTIGALEGAHFLATGNLESLSEQQLVDCDRECDPEEYDACDDGCNGGLMNNAFEYILKTGGVEREKDYPYTGRDRS
PCKFNESKIVASVSNFSVVSIDEDQIAANLVKNGPLAVGINAVFMQTYTAGVSCPFLCSGELDHGVLLVGYGSAGYSPIRF
KEKPYWILKNSWSKYWGEHGYYRICRGQNMCGVDSMVSSVVAAIQTTSQ*
>VvRD19-4
MGTVSRSALLFLIPTLLFSAAVSDISSDESDDLLIRQVVPEGDDLLSAEHQFGLFKAKFGKTYSTVEEHDYRFSVFEANLRR
ARRHQLLDPSAVHGVTRFSDLTPDEFRRDYLGLKPLRLPADAQKAPILPTNDLPTDFDWRDHGAVTPVKDQGSCGSCWS
FSAIGALEGAHFLTTGNLISMSEQQLVDCDHECDPEEYGACDQGCNGGLMTSAFEYILKAGGVEREETYPYIGSDRGSCK
FNKSQIVASVSNFSVVSLDEDQIAANMVKNGPLAVGINAVFMQTYMKGVSCPYICSRNLDHGVVLVGYGSAGYAPIRFKE
KPYWIIKNSWGESWGEDGYYKICRGHNACGVDSMVSTVAAIQTTTQ*
>VvRD19-5
MGGGLTCALGVAALLTCALAASAISLHEHDTPWDPNIVQVTDGHSHRKFGVDGVLGTEKEFRMFMEKYGKEYSSREEY
VHRLGIFAKNMVRAAEHQALDPTALHGVTPFSDLSEEEFERMFTGVVGRPHMKGGVAETAAALEVDGLPESFDWREKG
AVTEVKMQGTCGSCWAFSTTGAVEGAHFISTKKLLTLSEQQLVDCDHMCDIRDKTACDSGCEGGLMTNAYKYLIEAGGL
EEESSYPYTGKHGECKFKPDRVAVRVVNFTEVPINENQIAANLVCHGPLAVGLNAIFMQTYIGGVSCPLICPKRWINHGVL
LVGYGAKGYSILRFGYKPYWIIKNSWGKRWGEHGYYRLCRGHGMCGMNTMVSAVVTQTS*
>VvSAG12-6
MENSEPLVIHSKFPPTLGLRSETTSFRHQNVTRIPSTMDWRKKRTVTHIKNQLQCGGCWAFSAVAAMEGIAKLQTSKSISL
SEQELVDCDIFGSNIGCEGGCMDDAFKFIIQNRGLNSEARYLYKGVEGHCNKKKESSRAARINDYENMPEFSEKALLKVV
AHQPISVAIDAGGSAFQFYEIGIIT*
>VvCTB1
MAMNQLCLATILLLLGAISTFHPEVVALKSVSQLKFNTKILQESMVELINANPKAGWKAAMNPRFSNYSVGQFMHLLGV
KPTLQKDLEGVPVITHPKTLKLPKHFDARTAWPQCSTIGKILGRLLDSFSSYFDDFFCFGCTDALYFSYHLLVPFYIKDQGH
CGSCWAFGAVESLSDRFCIHFGMNISLSVNDLLACCGFLCGSGCDGGYPLYAWRYFIHHGVVTEECDPYFDATGCSHPGC
EPGYPTPKCVRKCTDENQLWRKAKRYGQSAYRISSDPYQIMAEVYKNGPVEVAFTVYEDFAHYESGVYRYTTGDVMGG
HAVKLIGWGTTDDGEDYWILANQWNRNWGDDGYFMIRRGVNECGIEEGVVAGLPSSKNLMIGDFESVDADRHVSI*
