# Supplementary material for: Characterization of the Populus PLCP Gene Family and the Function of PtrXCP2A in Wood Formation
Source: Plants (Basel). 2026 Oct 16;15(20):3174. doi: 10.3390/plants15203174 (PMC13708765; doi:10.3390/plants15203174)
Supplement: Supplementary file 1 [file plants-15-03174-s001.zip › Table S5.pdf]

Table S5. The primers used in this study

Purpose	Primer Name	Sequence (5'-3')
Primers for promoter vector construction		
ProXCP1	ProXCP1-F	CACCGTGTGACTTATAGGTTGTGC
	ProXCP1-R	GGAGATTGAGAGATTAGACAG
ProXCP2A	ProXCP2A-F	CACCTAGCTCCTAGCATCATCTGACC
	ProXCP2A-R	TTTAGCTGTTAATTGGATGGATGG
ProCEP1C	ProCEP1C-F	CACCGGATGCTCTCGCATGAGTTAC
	ProCEP1C-R	TTGCTAAGCTAGAACTCAACAC
Primers for overexpression vector construction		
pGWB10-XCP2A-FLAG (*ProXCP2A::XCP2A*)	XCP2A-PC-mid-F	CCATCCAATTAACAGCTAAAATGGCTCTCTCCTCAC
	XCP2A-PC-mid-R	GTGAGGAGAGAGCCATTTTAGCTGTTAATTGGATGG
	XCP2A-F	CACCATGGCTCTCTCCTCACTTTCTTTG
	XCP2A-R	CTTCTTTTTAGTGGGATAAGAAGCC
Primers used to identify transgenic plants		
GUS	GUS-F	GGGCGAACAGTTCCTGATTAACC
	GUS-R	CAGTACCTTCTCTGCCGTTTCCA
Hyg	Hyg-F	GAGCTTGTCGATCGACAGAT
	Hyg-R	CATATGCGCGATTGCTGATC
Kana	Kana-F	ATGATTGAACAAGATGGATTGCACG
	Kana-R	GGCAGGAGCAAGGTGAGATGACA
Primers used for RT-qPCR		
PtrXCP1	RT-XCP1-F	TGTGGCAGTTGTTGGGCA
	RT-XCP1-R	GAGGCCCCCATTACAGCC

PtrXCP2A	RT-XCP2A-F	CCAACCAGCCCCTCAGTG
	RT-XCP2A-R	ATACCCAACGGCTGCCAC
PtrXCP2B	RT-XCP2B-F	GCACTTGCAAACCAGCCC
	RT-XCP2B-R	ATCCAACGGCTGCCACTC
PtrRD19A	RT-RD19A-F	CCGCAGAGCAGCATCACT
	RT-RD19A-R	CGTGACTCGCTGTTGGGT
PtrRD19B	RT-RD19B-F	ATTGGACCCAACGGCGAG
	RT-RD19B-R	CTCAACCTGCGCAACCCT
PtrRD19C	RT-RD19C-F	GCTTGCGAGCCTGAGTGA
	RT-RD19C-R	CCAGAGTCACAAGCGCCA
PtrRD19E	RT-RD19E-F	GGCGCCGATCCTTCCTAC
	RT-RD19E-R	ACCAGCACGATCCACACG
PtrRD21A	RT-RD21A-F	AGCTGCTGCCCACATGAG
	RT-RD21A-R	GCGTCGCAATGCCTTCAC
PtrRDL1B	RT-RDL1B-F	GCCATGTTTCTGGGCACG
	RT-RDL1B-R	TTCCGGCAGCTTGTCACC
PtrALP2	RT-ALP2-F	TCCTCCACCTCTCGCTCC
	RT-ALP2-R	CAGCGACACGAGCCATGA
PtrCEP1C	RT-CEP1C-F	GAGTGGCAGTTGTGGGCT
	RT-CEP1C-R	GCCTCGCTGCATCCTGAT
PtrCEP1D	RT-CEP1D-F	GGAGCACCGCTGGGAAAT
	RT-CEP1D-R	ACGGCGCCTTTCTTCCTC
PtrPAL3	RT-PtrPAL3-F	AGCAGCTGGCATTGAATCTG
	RT-PtrPAL3-R	GCAAGGGGTCCGTTTCATGC

PtrPAL4	RT-PtrPAL4-F	ACGTTGGTGTCATGGTGGAG
	RT-PtrPAL4-R	GTGATAGCTTCCAGCATCTC
PtrC4H2	RT-PtrC4H2-F	AGGAGGAGACTGCAACTGAT
	RT-PtrC4H2-R	TTGCACATTTCAAGCCTTCA
Ptr4CL3	RT-Ptr4CL3-F	ACTAGCCCATCCAGAGATATCCGA
	RT-Ptr4CL3-R	TCATCTTCGGTGGCCTGAGACTTT
Ptr4CL5	RT-Ptr4CL5-F	GTGATCATGCTCATCCTGCCAAGT
	RT-Ptr4CL5-R	TTGGCAGCAGTAGTAATGGCACCT
PtrHCT1	RT-PtrHCT1-F	AGGTGACCTATTTCAAATGC
	RT-PtrHCT1-R	ATCAGGAAGTCCGCGTGCCT
PtrCCoAOMT2	RT-PtrCCoAOMT2-F	CCTTCCAACGCCAGGAAAGAGAGTA
	RT-PtrCCoAOMT2-R	GTGGCCAACTTCTTGATGCCTTCCG
PtrCCR2	RT-PtrCCR2-F	ATGTTGTCATTGATGAGTCT
	RT-PtrCCR2-R	GCGGAAGGCGTCTCAAAGAC
PtrCAld5H1	RT-PtrCAld5H1-F	AATCCAATATAGGCAAGCCTGTGAACG
	RT-PtrCAld5H1-R	ATTTTTGGCCCCAAAAGCTGCTCTA
PtrCAld5H2	RT-PtrCAld5H2-F	AAGCCAATATAGGCAAGCCTGTGAATC
	RT-PtrCAld5H2-R	ATTTTTAGCCCCGAAAGCTGCTCTG
PtrLAC3	RT-PtrLAC3-F	ATACAGCCTGGGCAAAGTTA
	RT-PtrLAC3-R	TTAGTGAAAGTAGCATCCAC
PtrCesA4	RT-PtrCesA4-F	GAGTTAAGGAAGATGGAGAGGTGT
	RT-PtrCesA4-R	TGCACTGAGGACAGGACTGGTTGC
PtrCesA7A	RT-PtrCesA7A-F	TCGCCTTTCTCTCAGATACGAACG
	RT-PtrCesA7A-R	TTACCCGTAACAAGAGGGGGTTCC

PtrCesA7B	RT-PtrCesA7B-F	CCCCTCTAGTCACGGGCAACACAC
	RT-PtrCesA7B-R	AAGGTGCACATTGAAGCACCATCG
PtrCesA8A	RT-PtrCesA8A-F	GTTGGCCTCTGTCTTCTCTCTTGTC
	RT-PtrCesA8A-R	CAATCTATAGAAATGCAGGTTTCAC
PtrCesA8B	RT-PtrCesA8B-F	GTTGGCCTCTGTCTTCTCTCTTGTT
	RT-PtrCesA8B-R	CAATCAATGGAAATGCAGGTCTCCG
PtrGT47A-1	RT-PtrGT47A-1-F	TCTCACACCCATGGGCT
	RT-PtrGT47A-1-R	AACGCTGGAGTAACGGG
PtrGT47C	RT-PtrGT47C-F	GTGGGACCCAGACGTTAGGC
	RT-PtrGT47C-R	CTATTGGTTTGGAAGCCTCACG
PtrCSLA1	RT-PtrCSLA1-F	ACATATTCCTCTTCCTCCAATCC
	RT-PtrCSLA1-R	CAAGAAAGATTCTTGCTAAGAGTG
PtrGAUT12-A	RT-PtrGAUT12-A-F	CCCCTGTGGGCTAAGTACATC
	RT-PtrGAUT12-A-R	TCACGATCGCAGTCAACATTTTC
PtrMYB021	RT-PtrMYB021-F	CCTTAACAGTGGTGATCATGGTTT
	RT-PtrMYB021-R	TTACTAGTGATGTTGCAGTTTTTGT
PtrMYB074	RT-PtrMYB074-F	CATAAGTTAGAGTCGGTGTTTCA
	RT-PtrMYB074-R	CAGCGACGTTAACGGATAGCTAT
PtrActin2	RT-PtrActin2-F	AACATGGGATTGTTAGCAACTGG
	RT-PtrActin2-R	TCCATCACCAGAATCCAGCACA
